# Supplementary material for: Molecular Characterization and Expression Profiling of Nuclear Receptor Gene Families in Oriental Fruit Fly, Bactrocera Dorsalis (Hendel)
Source: Insects. 2020 Feb 16;11(2):126. doi: 10.3390/insects11020126 (PMC7074177; doi:10.3390/insects11020126)
Supplement: Supplementary file 1 [file insects-11-00126-s001.zip › Table S1 Sequences from Drosophila melanogaster that were used to identify NR families in Bactrocera dorsalis.doc]

**Table S1 Sequences from *Drosophila melanogaster* that were used to identify NR families in *Bactrocera dorsalis*.**

**>DmKNRL—CG4761**

MMNQDNPYAMNQTCKVCGEPAAGFHFGAFTCEGCKSFFGRSYNNLSSISDCKNNGECIINKKNRTACKACRLKKCLMVGMSKSGSRYGRRSNWFKIHCLLQEQQQQAVAAMAAHHNSQQAGGGSSGGSGGGQGMPNGVKGMSGVPPPAAAAAALGMLGHPGGYPGLYAVANAGGSSRSKEELMMLGLDGSVEYGSHKHPVVASPSVSSPDSHNSDSSVEVSSVRGNPLLHLGGKSNSGGSSSGADGSHSGGGGGGGGGVTPGRPPQMRKDLSPFLPLPFPGLASMPVMPPPAFLPPSHLLFPGYHPALYSHHQGLLKPTPEQQQAAVAAAAVQHLFNSSGAGQRFAPGTSPFANHQQHHKEEDQPAPARSPSTHANNNHLLTNGGAADELTKRFYLDAVLKSQQQSPPPTTKLPPHSKQDYSISALVTPNSESGRERVKSRQNEEDDEARADGIIDGAEHDDEEEDLVVSMTPPHSPAQQEERTPAGEDPRPSPGQDNPIDLSMKTTGSSLSSKSSSPEIEPETEISSDVEKNDTDDDDEDLKVTPEEEISVRETADPEIEEDHSSTTETAKTSIENTHNNNNSISNNNNNNNNNNNSILSDSEASETIKRKLDELIEASSENGKRLRLEAPVKVATSNALDLTTKV

**>DmEGON—CG7383**

MNQLCKVCGEPAAGFHFGAFTCEGCKSFFGRTYNNIAAIAGCKHNGDCVINKKNRTACKACRLRKCLLVGMSKSGSRYGRRSNWFKIHCLLQEQQTTSGLGGGSSVGSGSGGGVSSASLEQLARLQQASNQARQTYQDKTNPCIKSATATTSPRIEGAAVGTGIGGGASPSFLQAAKLHHQRQLKLDSRLSNTPSDSGASSAGDPNEDGVTSVLGGQIATPSSTNATSLPKLDLRHPNFPATSEPDADMQRQRHQELLEIFRSHSEPLYSSFAPFSHLPPVLLAAGVPQLPIFKDQFKAELLFPTTSSPELEEPIDLSFRSRADPASPMAHNSNSPSLSEPAAASHCLGESTNFVRKSTPLDLTLVRSQTLTG

**>DmKNI—CG4717**

MVFNLMNQTCKVCGEPAAGFHFGAFTCEGCKSFFGRSYNNISTISECKNEGKCIIDKKNRTTCKACRLRKCYNVGMSKGGSRYGRRSNWFKIHCLLQEHEQAAAAAGKAPPLAGGVSVGGAPSASSPVGSPHTPGFGDMAAHLHHHHQQQQQQQVPRHPHMPLLGYPSYLSDPSAALPFFSMMGGVPHQSPFQLPPHLLFPGYHASAAAAAASAADAAYRQEMYKHRQSVDSVESQNRFSPASQPPVVQPTSSARQSPIDVCLEEDVHSVHSHQSSASLLHPIAIRATPTTPTSSSPLSFAAKMQSLSPVSVCSIGGETTSVVPVHPPTVSAQEGPMDLSMKTSRSSVHSFNDSGSEDQEVEVAPRRKFYQLEAECLTTSSSSSSHSAAHSPNTTTAHAEVKRQKLGGAEATHFGGFAVAHNAASAMRGIFVCV

**>DmE75—CG8127**

MEAVQAAAAATSSGGSSGSVPGSGSGSASKLIKTEPIDFEMLHLEENERQQDIEREPSSSNSNSNSNSLTPQRYTHVQVQTVPPRQPTGLTTPGGTQKVILTPRVEYVQQRATSSTGGGMKHVYSQQQGTAASRSAPPETTALLTTTSGTPQIIITRTLPSNQHLSRRHSASPSALHHYQQQQPQRQQSPPPLHHQQQQQQQHVRVIRDGRLYDEATVVVAARRHSVSPPPLHHHSRSAPVSPVIARRGGAAAYMDQQYQQRQTPPLAPPPPPPPPPPPPPPPQQQQQQYISTGVPPPTAAARKFVVSTSTRHVNVIASNHFQQQQQQHQAQQHQQQHQQHQQHQQHVIASVSSSSSSSAIGSGGSSSSHIFRTPVVSSSSSSNMHHQQQQQQQQSSLGNSVMRPPPPPPPPKVKHASSSSSGNSSSSNTNNSSSSSNGEEPSSSIPDLEFDGTTVLCRVCGDKASGFHYGVHSCEGCKGFFRRSIQQKIQYRPCTKNQQCSILRINRNRCQYCRLKKCIAVGMSRDAVRFGRVPKREKARILAAMQQSTQNRGQQRALATELDDQPRLLAAVLRAHLETCEFTKEKVSAMRQRARDCPSYSMPTLLACPLNPAPELQSEQEFSQRFAHVIRGVIDFAGMIPGFQLLTQDDKFTLLKAGLFDALFVRLICMFDSSINSIICLNGQVMRRDAIQNGANARFLVDSTFNFAERMNSMNLTDAEIGLFCAIVLITPDRPGLRNLELIEKMYSRLKGCLQYIVAQNRPDQPEFLAKLLETMPDLRTLSTLHTEKLVVFRTEHKELLRQQMWSMEDGNNSDGQQNKSPSGSWADAMDVEAAKSPLGSVSSTESADLDYGSPSSSQPQGVSLPSPPQQQPSALASSAPLLAATLSGGCPLRNRANSGSSGDSGAAEMDIVGSHAHLTQNGLTITPIVRHQQQQQQQQQIGILNNAHSRNLNGGHAMCQQQQQHPQLHHHLTAGAARYRKLDSPTDSGIESGNEKNECKAVSSGGSSSCSSPRSSVDDALDCSDAAANHNQVVQHPQLSVVSVSPVRSPQPSTSSHLKRQIVEDMPVLKRVLQAPPLYDTNSLMDEAYKPHKKFRALRHREFETAEADASSSTSGSNSLSAGSPRQSPVPNSVATPPPSAASAAAGNPAQSQLHMHLTRSSPKASMASSHSVLAKSLMAEPRMTPEQMKRSDIIQNYLKRENSTAASSTTNGVGNRSPSSSSTPPPSAVQNQQRWGSSSVITTTCQQRQQSVSPHSNGSSSSSSSSSSSSSSSSSTSSNCSSSSASSCQYFQSPHSTSNGTSAPASSSSGSNSATPLLELQVDIADSAQPLNLSKKSPTPPPSKLHALVAAANAVQRYPTLSADVTVTASNGGPPSAAASPAPSSSPPASVGSPNPGLSAAVHKVMLEA

**>DmE78—CG18023**

MDVYQIELEEQAQIRSKLLVETCVKHSSSEQQQLQVKQEDLIKDFTRDEEEQPSEEEAEEEDNEEDEEEEGEEEEEDEDEEALLPVVNFNANSDFNLHFFDTPEDSSTQGAYSEANSLESEQEEEKQTQQHQQQKQHHRDLEDCLSAIEADPLQLLHCDDFYRTSALAESVAASLSPQQQQQRQHTHQQQQQQQQQQQHPGQQQHQLNCTLSNGGGALYTISSVHQFGPASNHNTSSSSPSSSAAHSSPDSGCSSASSSGSSRSCGSSSASSSSSAVSSTISSGRSSNNSVVNPAATSSSVAHLNKEQQQQPLPTTQLQQQQQHQQQLQHPQQQQSFGLADSSSSNGSSNNNNGVSSKSFVPCKVCGDKASGYHYGVTSCEGCKGFFRRSIQKQIEYRCLRDGKCLVIRLNRNRCQYCRFKKCLSAGMSRDSVRYGRVPKRSRELNGAAASSAAAGAPASLNVDDSTSSTLHPSHLQQQQQQHLLQQQQQQQHQPQLQQHHQLQQQPHVSGVRVKTPSTPQTPQMCSIASSPSELGGCNSANNNNNNNNNSSSGNASGGSGVSVGVVVVGGHQQLVGGSMVGMAGMGTDAHQVGMCHDGLAGTANELTVYDVIMCVSQAHRLNCSYTEELTRELMRRPVTVPQNGIASTVAESLEFQKIWLWQQFSARVTPGVQRIVEFAKRVPGFCDFTQDDQLILIKLGFFEVWLTHVARLINEATLTLDDGAYLTRQQLEILYDSDFVNALLNFANTLNAYGLSDTEIGLFSAMVLLASDRAGLSEPKVIGRARELVAEALRVQILRSRAGSPQALQLMPALEAKIPELRSLGAKHFSHLDWLRMNWTKLRLPPLFAEIFDIPKADDEL

**>DmHR3—CG33183**

MDRPRDLLLIQQAKPAGGSKSSSGGPTYIGTDAATAAAVANLRSKVPSISIIPLPVSTTIVARSGSGASGTGGSGGAPPALPQLIQSSRSVSTKSTGTSPPLAFITEGSGTSGAPALTLLPRPQQQHQYQQTDKCTVLKLDVLKQQSGVAANSSSNTYLQIEQQLSELEAADEEEENMASLLGSAPPAQILANQPIIVKIEPTQSFHIVDEGDTRVLSLPLSDADKLGASWIDLKDIAGLQAGGGATLLDVCFEQANEDGTIIATVQPDLENELEAELKAEGEPEDETEPEPPAPKRLATTRPAQSRPQQQQQQQQQQVKFLSDPPALARSSSFSSLSSFSSISNISSVCKNMASNTSQEGSLKRTKERTPPPMPPLTTHKPAATTTATSATSAAAATSAATASATATSARENSREHSSSSSGSNGAMTAQIEIIPCKVCGDKSSGVHYGVITCEGCKGFFRRSQSSVVNYQCPRNKQCVVDRVNRNRCQYCRLQKCLKLGMSRDAVKFGRMSKKQREKVEDEVRFHRAQMRAQSDAAPDSSVYDTQTPSSSDQLHHNNYNSGGYSNNEVGYGSPYGYSASVTPQQTMQYDISADYVDSTTYEPRSTIIDPEFISHADGDINDVLIKTLAEAHANTNTKLEAVHDMFRKQPDVSRILYYKNLGQEELWLDCAEKLTQMIQNIIEFAKLIPGFMRLSQDDQILLLKTGSFELAIVRMSRLLDLSQNAVLYGDVMLPQEAFYTSDSEEMRLVSRIFQTAKSIAELKLTETELALYQSLVLLWPERNGVRGNTEIQRLFNLSMNAIRQELETNHAPLKGDVTVLDTLLNNIPNFRDISILHMESLSKFKLQHPNVVFPALYKELFSIDSQQDLT

**>DmECR—CG1765**

MKRRWSNNGGFMRLPEESSSEVTSSSNGLVLPSGVNMSPSSLDSHDYCDQDLWLCGNESGSFGGSNGHGLSQQQQSVITLAMHGCSSTLPAQTTIIPINGNANGNGGSTNGQYVPGATNLGALANGMLNGGFNGMQQQIQNGHGLINSTTPSTPTTPLHLQQNLGGAGGGGIGGMGILHHANGTPNGLIGVVGGGGGVGLGVGGGGVGGLGMQHTPRSDSVNSISSGRDDLSPSSSLNGYSANESCDAKKSKKGPAPRVQEELCLVCGDRASGYHYNALTCEGCKGFFRRSVTKSAVYCCKFGRACEMDMYMRRKCQECRLKKCLAVGMRPECVVPENQCAMKRREKKAQKEKDKMTTSPSSQHGGNGSLASGGGQDFVKKEILDLMTCEPPQHATIPLLPDEILAKCQARNIPSLTYNQLAVIYKLIWYQDGYEQPSEEDLRRIMSQPDENESQTDVSFRHITEITILTVQLIVEFAKGLPAFTKIPQEDQITLLKACSSEVMMLRMARRYDHSSDSIFFANNRSYTRDSYKMAGMADNIEDLLHFCRQMFSMKVDNVEYALLTAIVIFSDRPGLEKAQLVEAIQSYYIDTLRIYILNRHCGDSMSLVFYAKLLSILTELRTLGNQNAEMCFSLKLKNRKLPKFLEEIWDVHAIPPSVQSHLQITQEENERLERAERMRASVGGAITAGIDCDSASTSAAAAAAQHQPQPQPQPQPSSLTQNDSQHQTQPQLQPQLPPQLQGQLQPQLQPQLQTQLQPQIQPQPQLLPVSAPVPASVTAPGSLSAVSTSSEYMGGSAAIGPITPATTSSITAAVTASSTTSAVPMGNGVGVGVGVGGNVSMYANAQTAMALMGVALHSHQEQLIGGVAVKSEHSTTA

**>DmHR96—CG11783**

MSPPKNCAVCGDKALGYNFNAVTCESCKAFFRRNALAKKQFTCPFNQNCDITVVTRRFCQKCRLRKCLDIGMKSENIMSEEDKLIKRRKIETNRAKRRLMENGTDACDADGGEERDHKAPADSSSSNLDHYSGSQDSQSCGSADSGANGCSGRQASSPGTQVNPLQMTAEKIVDQIVSDPDRASQAINRLMRTQKEAISVMEKVISSQKDALRLVSHLIDYPGDALKIISKFMNSPFNALTVFTKFMSSPTDGVEIISKIVDSPADVVEFMQNLMHSPEDAIDIMNKFMNTPAEALRILNRILSGGGANAAQQTADRKPLLDKEPAVKPAAPAERADTVIQSMLGNSPPISPHDAAVDLQYHSPGVGEQPSTSSSHPLPYIANSPDFDLKTFMQTNYNDEPSLDSDFSINSIESVLSEVIRIEYQAFNSIQQAASRVKEEMSYGTQSTYGGCNSAANNSQPHLQQPICAPSTQQLDRELNEAEQMKLRELRLASEALYDPVDEDLSALMMGDDRIKPDDTRHNPKLLQLINLTAVAIKRLIKMAKKITAFRDMCQEDQVALLKGGCTEMMIMRSVMIYDDDRAAWKVPHTKENMGNIRTDLLKFAEGNIYEEHQKFITTFDEKWRMDENIILIMCAIVLFTSARSRVIHKDVIRLEQNSYYYLLRRYLESVYSGCEARNAFIKLIQKISDVERLNKFIINVYLNVNPSQVEPLLREIFDLKNH

**>DmHNF4—CG9310**

MVRKSGRVKISSRDRVAVGNILLRGKVGGGRVAVAAAEEAEAGRRRRRRDSSASRTASSDESESHIMHADALASAYPAASQPHSPIGLALSPNGGGLGLSNSSNQSSENFALCNGNGNAGSAGGGSASSGSNNNNSMFSPNNNLSGSGSGTNSSQQQLQQQQQQQSPTVCAICGDRATGKHYGASSCDGCKGFFRRSVRKNHQYTCRFARNCVVDKDKRNQCRYCRLRKCFKAGMKKEAVQNERDRISCRRTSNDDPDPGNGLSVISLVKAENESRQSKAGAAMEPNINEDLSNKQFASINDVCESMKQQLLTLVEWAKQIPAFNELQLDDQVALLRAHAGEHLLLGLSRRSMHLKDVLLLSNNCVITRHCPDPLVSPNLDISRIGARIIDELVTVMKDVGIDDTEFACIKALVFFDPNAKGLNEPHRIKSLRHQILNNLEDYISDRQYESRGRFGEILLILPVLQSITWQMIEQIQFAKIFGVAHIDSLLQEMLLGGELADNPLPLSPPNQSNDYQSPTHTGNMEGGNQVNSSLDSLATSGGPGSHSLDLEVQHIQALIEANSADDSFRAYAASTAAAAAAAVSSSSSAPASVAPASISPPLNSPKSQHQHQQHATHQQQQESSYLDMPVKHYNGSRSGPLPTQHSPQRMHPYQRAVASPVEVSSGGGGLGLRNPADITLNEYNRSEGSSAEELLRRTPLKIRAPEMLTAPAGYGTEPCRMTLKQEPETGY

**>DmUSP—CG4380**

MDNCDQDASFRLSHIKEEVKPDISQLNDSNNSSFSPKAESPVPFMQAMSMVHVLPGSNSASSNNNSAGDAQMAQAPNSAGGSAAAAVQQQYPPNHPLSGSKHLCSICGDRASGKHYGVYSCEGCKGFFKRTVRKDLTYACRENRNCIIDKRQRNRCQYCRYQKCLTCGMKREAVQEERQRGARNAAGRLSASGGGSSGPGSVGGSSSQGGGGGGGVSGGMGSGNGSDDFMTNSVSRDFSIERIIEAEQRAETQCGDRALTFLRVGPYSTVQPDYKGAVSALCQVVNKQLFQMVEYARMMPHFAQVPLDDQVILLKAAWIELLIANVAWCSIVSLDDGGAGGGGGGLGHDGSFERRSPGLQPQQLFLNQSFSYHRNSAIKAGVSAIFDRILSELSVKMKRLNLDRRELSCLKAIILYNPDIRGIKSRAEIEMCREKVYACLDEHCRLEHPGDDGRFAQLLLRLPALRSISLKCQDHLFLFRITSDRPLEELFLEQLEAPPPPGLAMKLE

**>DmHR78—CG7199**

MDGVKVETFIKSEENRAMPLIGGGSASGGTPLPGGGVGMGAGASATLSVELCLVCGDRASGRHYGAISCEGCKGFFKRSIRKQLGYQCRGAMNCEVTKHHRNRCQFCRLQKCLASGMRSDSVQHERKPIVDRKEGIIAAAGGSSTSGGGNGSSTYLSGKSGYQQGRGKGHSVKAESAATPPVHSAPATAFNLNENIFPMGLNFAELTQTLMFATQQQQQQQQQHQQSGSYSPDIPKADPEDDEDDSMDNSSTLCLQLLANSASNNNSQHLNFNAGEAPTALPTTSTMGLIQSSLDMRVIHKGLQILQPIQNQLERNGNLSVKPECDSEAEDSGTEDAVDAELEHMELDFECGGNRSGGSDFAINEAVFEQDLLTDVQCAFHVQPPTLVHSYLNIHYVCETGSRIIFLTIHTLRKVPVFEQLEAHTQVKLLRGVWPALMAIALAQCQGQLSVPTIIGQFIQSTRQLADIDKIEPLKISKMANLTRTLHDFVQELQSLDVTDMEFGLLRLILLFNPTLLQQRKERSLRGYVRRVQLYALSSLRRQGGIGGGEERFNVLVARLLPLSSLDAEAMEELFFANLVGQMQMDALIPFILMTSNTSGL

**>DmTLL—CG1378**

MQSSEGSPDMMDQKYNSVRLSPAASSRILYHVPCKVCRDHSSGKHYGIYACDGCAGFFKRSIRRSRQYVCKSQKQGLCVVDKTHRNQCRACRLRKCFEVGMNKDAVQHERGPRNSTLRRHMAMYKDAMMGAGEMPQIPAEILMNTAALTGFPGVPMPMPGLPQRAGHHPAHMAAFQPPPSAAAVLDLSVPRVPHHPVHQGHHGFFSPTAAYMNALATRALPPTPPLMAAEHIKETAAEHLFKNVNWIKSVRAFTELPMPDQLLLLEESWKEFFILAMAQYLMPMNFAQLLFVYESENANREIMGMVTREVHAFQEVLNQLCHLNIDSTEYECLRAISLFRKSPPSASSTEDLANSSILTGSGSPNSSASAESRGLLESGKVAAMHNDARSALHNYIQRTHPSQPMRFQTLLGVVQLMHKVSSFTIEELFFRKTIGDITIVRLISDMYSQRKI

**>DmHR51—CG16801**

MNKEENSSETRPSSQELHSPQRHCYTPPPAPMHGQAPPPTSTGVAPPTQPPPPHPAAPNVPNGRLLSWNHSAAAAAAAAAAQAAANSMNHSSAAEGSSMTRIKGQNLGLICVVCGDTSSGKHYGILACNGCSGFFKRSVRRKLIYRCQAGTGRCVVDKAHRNQCQACRLKKCLQMGMNKDAVQNERQPRNTATIRPETLREMEHGRALREAAVAVGVFGPPVLLSPPCYGSGLLPPPSLGSLPTGRLLHHNHLTSSMQLAANHMGAGSFPMFNAAGVHHSPKEKAYGMEMATSGNVSHSTNSSSNHSIDPSSPAPENAKEINIAGGSVSSVSSSSPTMENDNDDDSIDVTNDNEEPHAVSRSDSSFIMPQFMSPNLYTHQHETVYETSARLLFMAVKWAKNLPSFARLSFRDQVILLEESWSELFLLNAIQWCIPLDPTGCALFSVAEHCNNLENNANGDTCITKEELAADVRTLHEIFCKYKAVLVDPAEFACLKAIVLFRPETRGLKDPAQIENLQDQAHVMLSQHTKTQFTAQIARFGRLLLMLPLLRMISSHKIESIYFQRTIGNTPMEKVLCDMYKN

**>DmDSF—CG9019**

MGTAGDRLLDIPCKVCGDRSSGKHYGIYSCDGCSGFFKRSIHRNRIYTCKATGDLKGRCPVDKTHRNQCRACRLAKCFQSAMNKDAVQHERGPRKPKLHPQLHHHHHHAAAAAAAAHHAAAAHHHHHHHHHAHAAAAHHAAVAAAAASGLHHHHHAMPVSLVTNVSASFNYTQHISTHPPAPAAPPSGFHLTASGAQQGPAPPAGHLHHGGAGHQHATAFHHPGHGHALPAPHGGVVSNPGGNSSAISGSGPGSTLPFPSHLLHHNLIAEAASKLPGITATAVAAVVSSTSTPYASAAQTSSPSSNNHNYSSPSPSNSIQSISSIGSRSGGGEEGLSLGSESPRVNVETETPSPSNSPPLSAGSISPAPTLTTSSGSPQHRQMSRHSLSEATTPPSHASLMICASNNNNNNNNNNNNGEHKQSSYTSGSPTPTTPTPPPPRSGVGSTCNTASSSSGFLELLLSPDKCQELIQYQVQHNTLLFPQQLLDSRLLSWEMLQETTARLLFMAVRWVKCLMPFQTLSKNDQHLLLQESWKELFLLNLAQWTIPLDLTPILESPLIRERVLQDEATQTEMKTIQEILCRFRQITPDGSEVGCMKAIALFAPETAGLCDVQPVEMLQDQAQCILSDHVRLRYPRQATRFGRLLLLLPSLRTIRAATIEALFFKETIGNVPIARLLRDMYTMEPAQVDK

**>DmHR83—CG10296**

MSNFSACAVCGDQSSGKHYGVSCCDGCSCFFKRSVRRGSSYACIALVGNCVVDKARRNWCPSCRFQRCLAVGMNAAAVQEERGPRNQQVALYRTGRRQAPPSQAAPSPTPHSQALHFQILAQILVTCLRQAKANEQFALLDRCQQDAIFQVVWSEIFVLRASHWSLDISAMIDGCGDEQLKRLICEAHQLRADVLELNFMESLILCRKELAINAEYAVILGSHSKAALISLARYTLQQSNYLRFGQLLLGLRQLCLRRFDCALSCMFRSVVRDILKTL

**>DmSVP—CG11502**

MCASPSTAPGFFNPRPQSGAELSAFDIGLSRSMGLGVPPHSAWHEPPASLGGHLHAASAGPGTTTGSVATGGGGTTPSSVASQQSAVIKQDLSCPSLNQAGSGHHPGIKEDLSSSLPSANGGSAGGHHSGSGSGSGSGVNPGHGSDMLPLIKGHGQDMLTSIKGQPTGCGSTTPSSQANSSHSQSSNSGSQIDSKQNIECVVCGDKSSGKHYGQFTCEGCKSFFKRSVRRNLTYSCRGSRNCPIDQHHRNQCQYCRLKKCLKMGMRREAVQRGRVPPTQPGLAGMHGQYQIANGDPMGIAGFNGHSYLSSYISLLLRAEPYPTSRYGQCMQPNNIMGIDNICELAARLLFSAVEWAKNIPFFPELQVTDQVALLRLVWSELFVLNASQCSMPLHVAPLLAAAGLHASPMAADRVVAFMDHIRIFQEQVEKLKALHVDSAEYSCLKAIVLFTTGKLLDILYKDVPALLTKVSALLGKGSTASNDDVLAVVRDHLDELNRQEQESQAQQQAPLHLAAFMNCVAGVEAAVQQAEQAQVPTSSASASVSAPLVPSAGSAFSSCQAKSAGSEMDLLASLYAQAQATPPSSGGGDASGHNNSSGLGASLPTQSQSGSSSRNLTASPLSTSLATAPAPASASAPAPVPTSSVAQVPVPAPVPVTSSASSSSLGGGAYQTPSAAAAAAAMFHYQTPPRAAFGSAFDMFHHSTPFGVGVGHAHALAHSSGSGSASFGSPSYRYSPYSLAGSRWQL

**>DmERR—CG7404**

MKFYAGEGQGTNMSDGVSILHIKQEVDTPSASCFSPSSKSTATQSGTNGLKSSPSVSPERQLCSSTTSLSCDLHNVSLSNDGDSLKGSGTSGGNGGGGGGGTSGGNATNASAGAGSGSVRDELRRLCLVCGDVASGFHYGVASCEACKAFFKRTIQGNIEYTCPANNECEINKRRRKACQACRFQKCLLMGMLKEGVRLDRVRGGRQKYRRNPVSNSYQTMQLLYQSNTTSLCDVKILEVLNSYEPDALSVQTPPPQVHTTSITNDEASSSSGSIKLESSVVTPNGTCIFQNNNNNDPNEILSVLSDIYDKELVSVIGWAKQIPGFIDLPLNDQMKLLQVSWAEILTLQLTFRSLPFNGKLCFATDVWMDEHLAKECGYTEFYYHCVQIAQRMERISPRREEYYLLKALLLANCDILLDDQSSLRAFRDTILNSLNDVVYLLRHSSAVSHQQQLLLLLPSLRQADDILRRFWRGIARDEVITMKKLFLEMLEPLAR

**>DmHR38—CG1864**

MMRDRLASLIVVKQEGGSNTSISHHQATAIKCEASLYTESSLFQEINNNSCYRQNLNAPTHQQSHTSHLQHAQQHQTHQQHPLLPPPLPTLPLIYPCRNLFPDGCDINHLACCSSSNSNSNCNSDSNSTSSSPGNSHFFANGNTCAAALTPAPPATEPRKIKPLGAGKLKVGKTDSNSDSNSNCDSRAAAAASTSATSATSATTLAATAAATAAAAEAGGAASAAAAAKISQVRLTNQATTSMLLLQPNSSFSSLSPFDNFSTQTASTTTTTSASAAGHHQHHNHLLHQQHHNQQQQQQQQQQQQQQQQQQQEHLQQQHQQQLVSPQQHLLKSETLLSHEEDQLISNLTDSSVVSHSELFSDLFFPSDSNNSLLSPTTSGYPDNPAEDLTSSIENLTKLTCLRDKRLSSIPEQQLSSEQEQQLCLLSLRSSSDPAIALHAQQQQQQQQQQQQQQQQHQQQQQHLQLQLISPIGGPLSCGSSLPSFQETYSLKYNSSSGSSPQQASSSSTAAPTPTDQVLTLKMDEDCFPPLSGGWSASPPAPSQLQQLHTLQSQAQMSHPNSSNNSSNNAGNSHNNSGGYNYHGHFNAINASANLSPSSSASSLYEYNGVSAADNFYGQQQQQQQQSYQQHNYNSHNGERYSLPTFPTISELAAATAAVEAAAAATVSSPSVGGPPPVRRASLPVQRTVSPAGSTAQSPKLAKITLNQRHSHAHAHALQLNSAPNSAASSPASADLQAGRLLQAPSQLCAVCGDTAACQHYGVRTCEGCKGFFKRTVQKGSKYVCLADKNCPVDKRRRNRCQFCRFQKCLVVGMVKEVVRTDSLKGRRGRLPSKPKSPQESPPSPPISLITALVRSHVDTTPDPSCLDYSHYEEQSMSEADKVQQFYQLLTSSVDVIKQFAEKIPGYFDLLPEDQELLFQSASLELFVLRLAYRARIDDTKLIFCNGTVLHRTQCLRSFGEWLNDIMEFSRSLHNLEIDISAFACLCALTLITERHGLREPKKVEQLQMKIIGSLRDHVTYNAEAQKKQHYFSRLLGKLPELRSLSVQGLQRIFYLKLEDLVPAPALIENMFVTTLPF

**>DmFTZ-F1—CG4059**

MDTFNVPMLAESSNTNYATEATSNHHHLQHQHQQQHSHQQQQQQQLLMPHHHKDQMLAAGSSPMLPFYSHLQLQQKDATATIGPAAAAAAVEAATTSANADNFSSLQTIDASQLDGGISLSGLCDRFFVASPNPHSNSNMTLMGTATAATTTTTNNNNNNNTNNNNNNNVEAKTVRPSNGNSVIIESVTMPSFANILFPTHRSANECIDPALLQKNPQNPNGNNSSIIVPPVEYHQLKPLEVNSSTSVSTSNFLSSTTAQLLDFEVQVGKDDGHISTTTTTGPGSGSASGSGSGSGSGSGSIARTIGTATPTTTTSMSNTANPTRSSLHSIEELAASSCAPRAASPNSNHTSSASTTPQQQQQQQHHMQSGNHSGSNLSSDDESMSEDEFGLEIDDNGGYQDTTSSHSQQSGGGGGGGGGNLLNGSSGGSSAGGGYMLLPQAASSSGNNGNPNAGHMSSGSVGNGSGGAGNGGAGGNSGPGNPMGGTSATPGHGGEVIDFKHLFEELCPVCGDKVSGYHYGLLTCESCKGFFKRTVQNKKVYTCVAERSCHIDKTQRKRCPYCRFQKCLEVGMKLEAVRADRMRGGRNKFGPMYKRDRARKLQVMRQRQLALQALRNSMGPDIKPTPISPGYQQAYPNMNIKQEIQIPQVSSLTQSPDSSPSPIAIALGQVNASTGGVIATPMNAGTGGSGGGGLNGPSSVGNGNSSNGSSNGNNNSSTGNGTSGGGGGNNAGGGGGGTNSNDGLHRNGGNGNSSCHEAGIGSLQNTADSKLCFDSGTHPSSTADALIEPLRVSPMIREFVQSIDDREWQTQLFALLQKQTYNQVEVDLFELMCKVLDQNLFSQVDWARNTVFFKDLKVDDQMKLLQHSWSDMLVLDHLHHRIHNGLPDETQLNNGQVFNLMSLGLLGVPQLGDYFNELQNKLQDLKFDMGDYVCMKFLILLNPSVRGIVNRKTVSEGHDNVQAALLDYTLTCYPSVNDKFRGLVNILPEIHAMAVRGEDHLYTKHCAGSAPTQTLLMEMLHAKRKG

**>DmHR39—CG8676**

MPNMSSIKAEQQSGPLGGSSGYQVPVNMCTTTVANTTTTLGSSAGGATGSRHNVSVTNIKCELDELPSPNGNMVPVIANYVHGSLRIPLSGHSNHRESDSEEELASIENLKVRRRTAADKNGPRPMSWEGELSDTEVNGGEELMEMEPTIKSEVVPAVAPPQPVCALQPIKTELENIAGEMQIQEKCYPQSNTQHHAATKLKVAPTQSDPINLKFEPPLGDNSPLLAARSKSSSGGHLPLPTNPSPDSAIHSVYTHSSPSQSPLTSRHAPYTPSLSRNNSDASHSSCYSYSSEFSPTHSPIQARHAPPAGTLYGNHHGIYRQMKVEASSTVPSSGQEAQNLSMDSASSNLDTVGLGSSHPASPAGISRQQLINSPCPICGDKISGFHYGIFSCESCKGFFKRTVQNRKNYVCVRGGPCQVSISTRKKCPACRFEKCLQKGMKLEAIREDRTRGGRSTYQCSYTLPNSMLSPLLSPDQAAAAAAAAAVASQQQPHQRLHQLNGFGGVPIPCSTSLPASPSLAGTSVKSEEMAETGKQSLRTGSVPPLLQEIMDVEHLWQYTDAELARINQPLSAFASGSSSSSSSSGTSSGAHAQLTNPLLASAGLSSNGENANPDLIAHLCNVADHRLYKIVKWCKSLPLFKNISIDDQICLLINSWCELLLFSCCFRSIDTPGEIKMSQGRKITLSQAKSNGLQTCIERMLNLTDHLRRLRVDRYEYVAMKVIVLLQSDTTELQEAVKVRECQEKALQSLQAYTLAHYPDTPSKFGELLLRIPDLQRTCQLGKEMLTIKTRDGADFNLLMELLRGEH

**>DmHR4—CG43934**

MPICRIMTLSRGPYSELDKMSLFQDLKLKRRKIDSRCSSDGESVADTSTSSPDLLAPMSPKMCDTGGAGAGGGGGHGGGVGGALSSLPLPTAVVTLTATSTIAPVSAAAATICSSSTVPTAASSNTGHNVCVATPPTAATTVVVSAGKAAAIKAEQKNQQQQQQQQQQQTEINSSSGPVSAAAASSVMSPPPTTTVRMSPAPPTDLSLPAGRRTPSNATPTRATPTPPHNNGNASSSCGTSNGGGSSRASPDSMDERPSTTTTTTTGGTQMSTNGIHGGASGDCMQNAASAECGVSNLSVIRSVKEERIAASPTKMLSYHQHQQQQQQQMQYQQHAPSPTQSKGLTQSQQHVSVLVTPSRIKSELSPQQQQHAQQMPPTSLQHLAHHPAYSTSVSTATSSVTSTSGALTTTTSSSAAALHASTPSPTPLPTPAAVAAAAVAAAAAHHPHLHHPPPPIVQTHHLHQQLTQPQLRKSSPPTGLGLPQQHLLNQSMQHLTQQQQLQLLQQAAAMSQRPTQMSPNAINSSPSARHQSSASSPHQHLLQQHHHHQQQQQQQQQQQHLRIKKEPAQLLAAAAGSLLGSSGGAQRHLHSPHHHHPSSPQQQQQQPPPQLPPQALGNIAQLIHPGSLQLRHPNRDAAILFRVKNDVHQQVAAAQLMQPGAAAAAAAAAQRMVWVSNARINGVKPEVIGGPLGGLRPGGPAQSPSPHHSSSSSSSQLSPQTPSQTPPRGTPTVIMGESCGVRTMVWGYEPAPPTAGPSPTHGGNNAASTPQHPHGQQQQQPPSSMSSLSQQSHHSSQSSLQSASSPSAGSITPTHTPGPSPQNNEEAAQLLLSLGQTRIQDVRPRPHQFRTPHALNMERLWAGDYSQLPPGQIHALNLSAQQQWGSTNATGMNRGLDAPHEPTDEDDQPLVCMICEDKATGLHYGIITCEGCKGFFKRTVQNRRVYTCVADGTCEITKAQRNRCQYCRFKKCIEQGMVLQAVREDRMPGGRNSGAVYNLYKVKYKKHKKSKQQQQQQQQSQQQHMHSPHHQSPLSPHHQQHLLSPQQHLHQHQQQLAAAAAVAAAAQHQHSKLLSATLGQSEMKPMFLGPSIKQELLQQQQQQHQQQQAAQLHSPHQQQLHSPHHTLHGSPHGALPPHSSASALHSPLSHGPPSLPPHTSSSSSSSSSASSGGSSSSSQQPSHHALHSPHHALAHAHHLQANASLAPPHAQQLHQQQQQQQQQQLHQQHASQLQENAIKLPSHLVNGTILKTALTNPSEIVHLRNRLDTAVSSSKDRQLSYEHAHSMIQTLIDCDAMEDIATLPHFSEFLEDKSEISEKLCNIGDSIVHKLVSWTKKLPFYLEIPVDIHTKLLTDKWHEILILTTAAYQALHGRRRTSSGGRSSAGAPPHSPASANHHHNHHHMSGSPHATSLNLPHQLHHQQMQAAATPPLQKEDPEFVDEVNGHLLTLQTCLTTLMGQPIAMEQLKLDVGHMVDKMTQITIMFRRIKLKMEEYVCLKVYILLNKEVELETIQERYVQVLRTYLQHSSPQNPQDRLTELLSHIPEIQAAASLLLESKMFYVPFVLNSASVR
