# Supplementary material for: Molecular Characterization and Expression Profiling of Nuclear Receptor Gene Families in Oriental Fruit Fly, Bactrocera Dorsalis (Hendel)
Source: Insects. 2020 Feb 16;11(2):126. doi: 10.3390/insects11020126 (PMC7074177; doi:10.3390/insects11020126)
Supplement: Supplementary file 1 [file insects-11-00126-s001.zip › Table S2 Sequences of 90 insect nuclear receptor (NR) proteins from five different species for the phylogenetic analysis.doc]

**Table S2** **Sequences of 90 nuclear receptor (NR) proteins from five insect species for the phylogenetic analysis**

***Drosophila melanogaster* (Dm)**

>DmKNRL—CG4761

MMNQDNPYAMNQTCKVCGEPAAGFHFGAFTCEGCKSFFGRSYNNLSSISDCKNNGECIINKKNRTACKACRLKKCLMVGMSKSGSRYGRRSNWFKIHCLLQEQQQQAVAAMAAHHNSQQAGGGSSGGSGGGQGMPNGVKGMSGVPPPAAAAAALGMLGHPGGYPGLYAVANAGGSSRSKEELMMLGLDGSVEYGSHKHPVVASPSVSSPDSHNSDSSVEVSSVRGNPLLHLGGKSNSGGSSSGADGSHSGGGGGGGGGVTPGRPPQMRKDLSPFLPLPFPGLASMPVMPPPAFLPPSHLLFPGYHPALYSHHQGLLKPTPEQQQAAVAAAAVQHLFNSSGAGQRFAPGTSPFANHQQHHKEEDQPAPARSPSTHANNNHLLTNGGAADELTKRFYLDAVLKSQQQSPPPTTKLPPHSKQDYSISALVTPNSESGRERVKSRQNEEDDEARADGIIDGAEHDDEEEDLVVSMTPPHSPAQQEERTPAGEDPRPSPGQDNPIDLSMKTTGSSLSSKSSSPEIEPETEISSDVEKNDTDDDDEDLKVTPEEEISVRETADPEIEEDHSSTTETAKTSIENTHNNNNSISNNNNNNNNNNNSILSDSEASETIKRKLDELIEASSENGKRLRLEAPVKVATSNALDLTTKV

>DmEGON—CG7383

MNQLCKVCGEPAAGFHFGAFTCEGCKSFFGRTYNNIAAIAGCKHNGDCVINKKNRTACKACRLRKCLLVGMSKSGSRYGRRSNWFKIHCLLQEQQTTSGLGGGSSVGSGSGGGVSSASLEQLARLQQASNQARQTYQDKTNPCIKSATATTSPRIEGAAVGTGIGGGASPSFLQAAKLHHQRQLKLDSRLSNTPSDSGASSAGDPNEDGVTSVLGGQIATPSSTNATSLPKLDLRHPNFPATSEPDADMQRQRHQELLEIFRSHSEPLYSSFAPFSHLPPVLLAAGVPQLPIFKDQFKAELLFPTTSSPELEEPIDLSFRSRADPASPMAHNSNSPSLSEPAAASHCLGESTNFVRKSTPLDLTLVRSQTLTG

>DmKNI—CG4717

MVFNLMNQTCKVCGEPAAGFHFGAFTCEGCKSFFGRSYNNISTISECKNEGKCIIDKKNRTTCKACRLRKCYNVGMSKGGSRYGRRSNWFKIHCLLQEHEQAAAAAGKAPPLAGGVSVGGAPSASSPVGSPHTPGFGDMAAHLHHHHQQQQQQQVPRHPHMPLLGYPSYLSDPSAALPFFSMMGGVPHQSPFQLPPHLLFPGYHASAAAAAASAADAAYRQEMYKHRQSVDSVESQNRFSPASQPPVVQPTSSARQSPIDVCLEEDVHSVHSHQSSASLLHPIAIRATPTTPTSSSPLSFAAKMQSLSPVSVCSIGGETTSVVPVHPPTVSAQEGPMDLSMKTSRSSVHSFNDSGSEDQEVEVAPRRKFYQLEAECLTTSSSSSSHSAAHSPNTTTAHAEVKRQKLGGAEATHFGGFAVAHNAASAMRGIFVCV

>DmE75—CG8127

MEAVQAAAAATSSGGSSGSVPGSGSGSASKLIKTEPIDFEMLHLEENERQQDIEREPSSSNSNSNSNSLTPQRYTHVQVQTVPPRQPTGLTTPGGTQKVILTPRVEYVQQRATSSTGGGMKHVYSQQQGTAASRSAPPETTALLTTTSGTPQIIITRTLPSNQHLSRRHSASPSALHHYQQQQPQRQQSPPPLHHQQQQQQQHVRVIRDGRLYDEATVVVAARRHSVSPPPLHHHSRSAPVSPVIARRGGAAAYMDQQYQQRQTPPLAPPPPPPPPPPPPPPPQQQQQQYISTGVPPPTAAARKFVVSTSTRHVNVIASNHFQQQQQQHQAQQHQQQHQQHQQHQQHVIASVSSSSSSSAIGSGGSSSSHIFRTPVVSSSSSSNMHHQQQQQQQQSSLGNSVMRPPPPPPPPKVKHASSSSSGNSSSSNTNNSSSSSNGEEPSSSIPDLEFDGTTVLCRVCGDKASGFHYGVHSCEGCKGFFRRSIQQKIQYRPCTKNQQCSILRINRNRCQYCRLKKCIAVGMSRDAVRFGRVPKREKARILAAMQQSTQNRGQQRALATELDDQPRLLAAVLRAHLETCEFTKEKVSAMRQRARDCPSYSMPTLLACPLNPAPELQSEQEFSQRFAHVIRGVIDFAGMIPGFQLLTQDDKFTLLKAGLFDALFVRLICMFDSSINSIICLNGQVMRRDAIQNGANARFLVDSTFNFAERMNSMNLTDAEIGLFCAIVLITPDRPGLRNLELIEKMYSRLKGCLQYIVAQNRPDQPEFLAKLLETMPDLRTLSTLHTEKLVVFRTEHKELLRQQMWSMEDGNNSDGQQNKSPSGSWADAMDVEAAKSPLGSVSSTESADLDYGSPSSSQPQGVSLPSPPQQQPSALASSAPLLAATLSGGCPLRNRANSGSSGDSGAAEMDIVGSHAHLTQNGLTITPIVRHQQQQQQQQQIGILNNAHSRNLNGGHAMCQQQQQHPQLHHHLTAGAARYRKLDSPTDSGIESGNEKNECKAVSSGGSSSCSSPRSSVDDALDCSDAAANHNQVVQHPQLSVVSVSPVRSPQPSTSSHLKRQIVEDMPVLKRVLQAPPLYDTNSLMDEAYKPHKKFRALRHREFETAEADASSSTSGSNSLSAGSPRQSPVPNSVATPPPSAASAAAGNPAQSQLHMHLTRSSPKASMASSHSVLAKSLMAEPRMTPEQMKRSDIIQNYLKRENSTAASSTTNGVGNRSPSSSSTPPPSAVQNQQRWGSSSVITTTCQQRQQSVSPHSNGSSSSSSSSSSSSSSSSSTSSNCSSSSASSCQYFQSPHSTSNGTSAPASSSSGSNSATPLLELQVDIADSAQPLNLSKKSPTPPPSKLHALVAAANAVQRYPTLSADVTVTASNGGPPSAAASPAPSSSPPASVGSPNPGLSAAVHKVMLEA

>DmE78—CG18023

MDVYQIELEEQAQIRSKLLVETCVKHSSSEQQQLQVKQEDLIKDFTRDEEEQPSEEEAEEEDNEEDEEEEGEEEEEDEDEEALLPVVNFNANSDFNLHFFDTPEDSSTQGAYSEANSLESEQEEEKQTQQHQQQKQHHRDLEDCLSAIEADPLQLLHCDDFYRTSALAESVAASLSPQQQQQRQHTHQQQQQQQQQQQHPGQQQHQLNCTLSNGGGALYTISSVHQFGPASNHNTSSSSPSSSAAHSSPDSGCSSASSSGSSRSCGSSSASSSSSAVSSTISSGRSSNNSVVNPAATSSSVAHLNKEQQQQPLPTTQLQQQQQHQQQLQHPQQQQSFGLADSSSSNGSSNNNNGVSSKSFVPCKVCGDKASGYHYGVTSCEGCKGFFRRSIQKQIEYRCLRDGKCLVIRLNRNRCQYCRFKKCLSAGMSRDSVRYGRVPKRSRELNGAAASSAAAGAPASLNVDDSTSSTLHPSHLQQQQQQHLLQQQQQQQHQPQLQQHHQLQQQPHVSGVRVKTPSTPQTPQMCSIASSPSELGGCNSANNNNNNNNNSSSGNASGGSGVSVGVVVVGGHQQLVGGSMVGMAGMGTDAHQVGMCHDGLAGTANELTVYDVIMCVSQAHRLNCSYTEELTRELMRRPVTVPQNGIASTVAESLEFQKIWLWQQFSARVTPGVQRIVEFAKRVPGFCDFTQDDQLILIKLGFFEVWLTHVARLINEATLTLDDGAYLTRQQLEILYDSDFVNALLNFANTLNAYGLSDTEIGLFSAMVLLASDRAGLSEPKVIGRARELVAEALRVQILRSRAGSPQALQLMPALEAKIPELRSLGAKHFSHLDWLRMNWTKLRLPPLFAEIFDIPKADDEL

>DmHR3—CG33183

MDRPRDLLLIQQAKPAGGSKSSSGGPTYIGTDAATAAAVANLRSKVPSISIIPLPVSTTIVARSGSGASGTGGSGGAPPALPQLIQSSRSVSTKSTGTSPPLAFITEGSGTSGAPALTLLPRPQQQHQYQQTDKCTVLKLDVLKQQSGVAANSSSNTYLQIEQQLSELEAADEEEENMASLLGSAPPAQILANQPIIVKIEPTQSFHIVDEGDTRVLSLPLSDADKLGASWIDLKDIAGLQAGGGATLLDVCFEQANEDGTIIATVQPDLENELEAELKAEGEPEDETEPEPPAPKRLATTRPAQSRPQQQQQQQQQQVKFLSDPPALARSSSFSSLSSFSSISNISSVCKNMASNTSQEGSLKRTKERTPPPMPPLTTHKPAATTTATSATSAAAATSAATASATATSARENSREHSSSSSGSNGAMTAQIEIIPCKVCGDKSSGVHYGVITCEGCKGFFRRSQSSVVNYQCPRNKQCVVDRVNRNRCQYCRLQKCLKLGMSRDAVKFGRMSKKQREKVEDEVRFHRAQMRAQSDAAPDSSVYDTQTPSSSDQLHHNNYNSGGYSNNEVGYGSPYGYSASVTPQQTMQYDISADYVDSTTYEPRSTIIDPEFISHADGDINDVLIKTLAEAHANTNTKLEAVHDMFRKQPDVSRILYYKNLGQEELWLDCAEKLTQMIQNIIEFAKLIPGFMRLSQDDQILLLKTGSFELAIVRMSRLLDLSQNAVLYGDVMLPQEAFYTSDSEEMRLVSRIFQTAKSIAELKLTETELALYQSLVLLWPERNGVRGNTEIQRLFNLSMNAIRQELETNHAPLKGDVTVLDTLLNNIPNFRDISILHMESLSKFKLQHPNVVFPALYKELFSIDSQQDLT

>DmECR—CG1765

MKRRWSNNGGFMRLPEESSSEVTSSSNGLVLPSGVNMSPSSLDSHDYCDQDLWLCGNESGSFGGSNGHGLSQQQQSVITLAMHGCSSTLPAQTTIIPINGNANGNGGSTNGQYVPGATNLGALANGMLNGGFNGMQQQIQNGHGLINSTTPSTPTTPLHLQQNLGGAGGGGIGGMGILHHANGTPNGLIGVVGGGGGVGLGVGGGGVGGLGMQHTPRSDSVNSISSGRDDLSPSSSLNGYSANESCDAKKSKKGPAPRVQEELCLVCGDRASGYHYNALTCEGCKGFFRRSVTKSAVYCCKFGRACEMDMYMRRKCQECRLKKCLAVGMRPECVVPENQCAMKRREKKAQKEKDKMTTSPSSQHGGNGSLASGGGQDFVKKEILDLMTCEPPQHATIPLLPDEILAKCQARNIPSLTYNQLAVIYKLIWYQDGYEQPSEEDLRRIMSQPDENESQTDVSFRHITEITILTVQLIVEFAKGLPAFTKIPQEDQITLLKACSSEVMMLRMARRYDHSSDSIFFANNRSYTRDSYKMAGMADNIEDLLHFCRQMFSMKVDNVEYALLTAIVIFSDRPGLEKAQLVEAIQSYYIDTLRIYILNRHCGDSMSLVFYAKLLSILTELRTLGNQNAEMCFSLKLKNRKLPKFLEEIWDVHAIPPSVQSHLQITQEENERLERAERMRASVGGAITAGIDCDSASTSAAAAAAQHQPQPQPQPQPSSLTQNDSQHQTQPQLQPQLPPQLQGQLQPQLQPQLQTQLQPQIQPQPQLLPVSAPVPASVTAPGSLSAVSTSSEYMGGSAAIGPITPATTSSITAAVTASSTTSAVPMGNGVGVGVGVGGNVSMYANAQTAMALMGVALHSHQEQLIGGVAVKSEHSTTA

>DmHR96—CG11783

MSPPKNCAVCGDKALGYNFNAVTCESCKAFFRRNALAKKQFTCPFNQNCDITVVTRRFCQKCRLRKCLDIGMKSENIMSEEDKLIKRRKIETNRAKRRLMENGTDACDADGGEERDHKAPADSSSSNLDHYSGSQDSQSCGSADSGANGCSGRQASSPGTQVNPLQMTAEKIVDQIVSDPDRASQAINRLMRTQKEAISVMEKVISSQKDALRLVSHLIDYPGDALKIISKFMNSPFNALTVFTKFMSSPTDGVEIISKIVDSPADVVEFMQNLMHSPEDAIDIMNKFMNTPAEALRILNRILSGGGANAAQQTADRKPLLDKEPAVKPAAPAERADTVIQSMLGNSPPISPHDAAVDLQYHSPGVGEQPSTSSSHPLPYIANSPDFDLKTFMQTNYNDEPSLDSDFSINSIESVLSEVIRIEYQAFNSIQQAASRVKEEMSYGTQSTYGGCNSAANNSQPHLQQPICAPSTQQLDRELNEAEQMKLRELRLASEALYDPVDEDLSALMMGDDRIKPDDTRHNPKLLQLINLTAVAIKRLIKMAKKITAFRDMCQEDQVALLKGGCTEMMIMRSVMIYDDDRAAWKVPHTKENMGNIRTDLLKFAEGNIYEEHQKFITTFDEKWRMDENIILIMCAIVLFTSARSRVIHKDVIRLEQNSYYYLLRRYLESVYSGCEARNAFIKLIQKISDVERLNKFIINVYLNVNPSQVEPLLREIFDLKNH

>DmHNF4—CG9310

MVRKSGRVKISSRDRVAVGNILLRGKVGGGRVAVAAAEEAEAGRRRRRRDSSASRTASSDESESHIMHADALASAYPAASQPHSPIGLALSPNGGGLGLSNSSNQSSENFALCNGNGNAGSAGGGSASSGSNNNNSMFSPNNNLSGSGSGTNSSQQQLQQQQQQQSPTVCAICGDRATGKHYGASSCDGCKGFFRRSVRKNHQYTCRFARNCVVDKDKRNQCRYCRLRKCFKAGMKKEAVQNERDRISCRRTSNDDPDPGNGLSVISLVKAENESRQSKAGAAMEPNINEDLSNKQFASINDVCESMKQQLLTLVEWAKQIPAFNELQLDDQVALLRAHAGEHLLLGLSRRSMHLKDVLLLSNNCVITRHCPDPLVSPNLDISRIGARIIDELVTVMKDVGIDDTEFACIKALVFFDPNAKGLNEPHRIKSLRHQILNNLEDYISDRQYESRGRFGEILLILPVLQSITWQMIEQIQFAKIFGVAHIDSLLQEMLLGGELADNPLPLSPPNQSNDYQSPTHTGNMEGGNQVNSSLDSLATSGGPGSHSLDLEVQHIQALIEANSADDSFRAYAASTAAAAAAAVSSSSSAPASVAPASISPPLNSPKSQHQHQQHATHQQQQESSYLDMPVKHYNGSRSGPLPTQHSPQRMHPYQRAVASPVEVSSGGGGLGLRNPADITLNEYNRSEGSSAEELLRRTPLKIRAPEMLTAPAGYGTEPCRMTLKQEPETGY

>DmUSP—CG4380

MDNCDQDASFRLSHIKEEVKPDISQLNDSNNSSFSPKAESPVPFMQAMSMVHVLPGSNSASSNNNSAGDAQMAQAPNSAGGSAAAAVQQQYPPNHPLSGSKHLCSICGDRASGKHYGVYSCEGCKGFFKRTVRKDLTYACRENRNCIIDKRQRNRCQYCRYQKCLTCGMKREAVQEERQRGARNAAGRLSASGGGSSGPGSVGGSSSQGGGGGGGVSGGMGSGNGSDDFMTNSVSRDFSIERIIEAEQRAETQCGDRALTFLRVGPYSTVQPDYKGAVSALCQVVNKQLFQMVEYARMMPHFAQVPLDDQVILLKAAWIELLIANVAWCSIVSLDDGGAGGGGGGLGHDGSFERRSPGLQPQQLFLNQSFSYHRNSAIKAGVSAIFDRILSELSVKMKRLNLDRRELSCLKAIILYNPDIRGIKSRAEIEMCREKVYACLDEHCRLEHPGDDGRFAQLLLRLPALRSISLKCQDHLFLFRITSDRPLEELFLEQLEAPPPPGLAMKLE

>DmDHR78—CG7199

MDGVKVETFIKSEENRAMPLIGGGSASGGTPLPGGGVGMGAGASATLSVELCLVCGDRASGRHYGAISCEGCKGFFKRSIRKQLGYQCRGAMNCEVTKHHRNRCQFCRLQKCLASGMRSDSVQHERKPIVDRKEGIIAAAGGSSTSGGGNGSSTYLSGKSGYQQGRGKGHSVKAESAATPPVHSAPATAFNLNENIFPMGLNFAELTQTLMFATQQQQQQQQQHQQSGSYSPDIPKADPEDDEDDSMDNSSTLCLQLLANSASNNNSQHLNFNAGEAPTALPTTSTMGLIQSSLDMRVIHKGLQILQPIQNQLERNGNLSVKPECDSEAEDSGTEDAVDAELEHMELDFECGGNRSGGSDFAINEAVFEQDLLTDVQCAFHVQPPTLVHSYLNIHYVCETGSRIIFLTIHTLRKVPVFEQLEAHTQVKLLRGVWPALMAIALAQCQGQLSVPTIIGQFIQSTRQLADIDKIEPLKISKMANLTRTLHDFVQELQSLDVTDMEFGLLRLILLFNPTLLQQRKERSLRGYVRRVQLYALSSLRRQGGIGGGEERFNVLVARLLPLSSLDAEAMEELFFANLVGQMQMDALIPFILMTSNTSGL

>DmTLL—CG1378

MQSSEGSPDMMDQKYNSVRLSPAASSRILYHVPCKVCRDHSSGKHYGIYACDGCAGFFKRSIRRSRQYVCKSQKQGLCVVDKTHRNQCRACRLRKCFEVGMNKDAVQHERGPRNSTLRRHMAMYKDAMMGAGEMPQIPAEILMNTAALTGFPGVPMPMPGLPQRAGHHPAHMAAFQPPPSAAAVLDLSVPRVPHHPVHQGHHGFFSPTAAYMNALATRALPPTPPLMAAEHIKETAAEHLFKNVNWIKSVRAFTELPMPDQLLLLEESWKEFFILAMAQYLMPMNFAQLLFVYESENANREIMGMVTREVHAFQEVLNQLCHLNIDSTEYECLRAISLFRKSPPSASSTEDLANSSILTGSGSPNSSASAESRGLLESGKVAAMHNDARSALHNYIQRTHPSQPMRFQTLLGVVQLMHKVSSFTIEELFFRKTIGDITIVRLISDMYSQRKI

>DmHR51—CG16801

MNKEENSSETRPSSQELHSPQRHCYTPPPAPMHGQAPPPTSTGVAPPTQPPPPHPAAPNVPNGRLLSWNHSAAAAAAAAAAQAAANSMNHSSAAEGSSMTRIKGQNLGLICVVCGDTSSGKHYGILACNGCSGFFKRSVRRKLIYRCQAGTGRCVVDKAHRNQCQACRLKKCLQMGMNKDAVQNERQPRNTATIRPETLREMEHGRALREAAVAVGVFGPPVLLSPPCYGSGLLPPPSLGSLPTGRLLHHNHLTSSMQLAANHMGAGSFPMFNAAGVHHSPKEKAYGMEMATSGNVSHSTNSSSNHSIDPSSPAPENAKEINIAGGSVSSVSSSSPTMENDNDDDSIDVTNDNEEPHAVSRSDSSFIMPQFMSPNLYTHQHETVYETSARLLFMAVKWAKNLPSFARLSFRDQVILLEESWSELFLLNAIQWCIPLDPTGCALFSVAEHCNNLENNANGDTCITKEELAADVRTLHEIFCKYKAVLVDPAEFACLKAIVLFRPETRGLKDPAQIENLQDQAHVMLSQHTKTQFTAQIARFGRLLLMLPLLRMISSHKIESIYFQRTIGNTPMEKVLCDMYKN

>DmDSF—CG9019

MGTAGDRLLDIPCKVCGDRSSGKHYGIYSCDGCSGFFKRSIHRNRIYTCKATGDLKGRCPVDKTHRNQCRACRLAKCFQSAMNKDAVQHERGPRKPKLHPQLHHHHHHAAAAAAAAHHAAAAHHHHHHHHHAHAAAAHHAAVAAAAASGLHHHHHAMPVSLVTNVSASFNYTQHISTHPPAPAAPPSGFHLTASGAQQGPAPPAGHLHHGGAGHQHATAFHHPGHGHALPAPHGGVVSNPGGNSSAISGSGPGSTLPFPSHLLHHNLIAEAASKLPGITATAVAAVVSSTSTPYASAAQTSSPSSNNHNYSSPSPSNSIQSISSIGSRSGGGEEGLSLGSESPRVNVETETPSPSNSPPLSAGSISPAPTLTTSSGSPQHRQMSRHSLSEATTPPSHASLMICASNNNNNNNNNNNNGEHKQSSYTSGSPTPTTPTPPPPRSGVGSTCNTASSSSGFLELLLSPDKCQELIQYQVQHNTLLFPQQLLDSRLLSWEMLQETTARLLFMAVRWVKCLMPFQTLSKNDQHLLLQESWKELFLLNLAQWTIPLDLTPILESPLIRERVLQDEATQTEMKTIQEILCRFRQITPDGSEVGCMKAIALFAPETAGLCDVQPVEMLQDQAQCILSDHVRLRYPRQATRFGRLLLLLPSLRTIRAATIEALFFKETIGNVPIARLLRDMYTMEPAQVDK

>DmHR83—CG10296

MSNFSACAVCGDQSSGKHYGVSCCDGCSCFFKRSVRRGSSYACIALVGNCVVDKARRNWCPSCRFQRCLAVGMNAAAVQEERGPRNQQVALYRTGRRQAPPSQAAPSPTPHSQALHFQILAQILVTCLRQAKANEQFALLDRCQQDAIFQVVWSEIFVLRASHWSLDISAMIDGCGDEQLKRLICEAHQLRADVLELNFMESLILCRKELAINAEYAVILGSHSKAALISLARYTLQQSNYLRFGQLLLGLRQLCLRRFDCALSCMFRSVVRDILKTL

>DmSVP—CG11502

MCASPSTAPGFFNPRPQSGAELSAFDIGLSRSMGLGVPPHSAWHEPPASLGGHLHAASAGPGTTTGSVATGGGGTTPSSVASQQSAVIKQDLSCPSLNQAGSGHHPGIKEDLSSSLPSANGGSAGGHHSGSGSGSGSGVNPGHGSDMLPLIKGHGQDMLTSIKGQPTGCGSTTPSSQANSSHSQSSNSGSQIDSKQNIECVVCGDKSSGKHYGQFTCEGCKSFFKRSVRRNLTYSCRGSRNCPIDQHHRNQCQYCRLKKCLKMGMRREAVQRGRVPPTQPGLAGMHGQYQIANGDPMGIAGFNGHSYLSSYISLLLRAEPYPTSRYGQCMQPNNIMGIDNICELAARLLFSAVEWAKNIPFFPELQVTDQVALLRLVWSELFVLNASQCSMPLHVAPLLAAAGLHASPMAADRVVAFMDHIRIFQEQVEKLKALHVDSAEYSCLKAIVLFTTGKLLDILYKDVPALLTKVSALLGKGSTASNDDVLAVVRDHLDELNRQEQESQAQQQAPLHLAAFMNCVAGVEAAVQQAEQAQVPTSSASASVSAPLVPSAGSAFSSCQAKSAGSEMDLLASLYAQAQATPPSSGGGDASGHNNSSGLGASLPTQSQSGSSSRNLTASPLSTSLATAPAPASASAPAPVPTSSVAQVPVPAPVPVTSSASSSSLGGGAYQTPSAAAAAAAMFHYQTPPRAAFGSAFDMFHHSTPFGVGVGHAHALAHSSGSGSASFGSPSYRYSPYSLAGSRWQL

>DmERR—CG7404

MKFYAGEGQGTNMSDGVSILHIKQEVDTPSASCFSPSSKSTATQSGTNGLKSSPSVSPERQLCSSTTSLSCDLHNVSLSNDGDSLKGSGTSGGNGGGGGGGTSGGNATNASAGAGSGSVRDELRRLCLVCGDVASGFHYGVASCEACKAFFKRTIQGNIEYTCPANNECEINKRRRKACQACRFQKCLLMGMLKEGVRLDRVRGGRQKYRRNPVSNSYQTMQLLYQSNTTSLCDVKILEVLNSYEPDALSVQTPPPQVHTTSITNDEASSSSGSIKLESSVVTPNGTCIFQNNNNNDPNEILSVLSDIYDKELVSVIGWAKQIPGFIDLPLNDQMKLLQVSWAEILTLQLTFRSLPFNGKLCFATDVWMDEHLAKECGYTEFYYHCVQIAQRMERISPRREEYYLLKALLLANCDILLDDQSSLRAFRDTILNSLNDVVYLLRHSSAVSHQQQLLLLLPSLRQADDILRRFWRGIARDEVITMKKLFLEMLEPLAR

>DmHR38—CG1864

MMRDRLASLIVVKQEGGSNTSISHHQATAIKCEASLYTESSLFQEINNNSCYRQNLNAPTHQQSHTSHLQHAQQHQTHQQHPLLPPPLPTLPLIYPCRNLFPDGCDINHLACCSSSNSNSNCNSDSNSTSSSPGNSHFFANGNTCAAALTPAPPATEPRKIKPLGAGKLKVGKTDSNSDSNSNCDSRAAAAASTSATSATSATTLAATAAATAAAAEAGGAASAAAAAKISQVRLTNQATTSMLLLQPNSSFSSLSPFDNFSTQTASTTTTTSASAAGHHQHHNHLLHQQHHNQQQQQQQQQQQQQQQQQQQEHLQQQHQQQLVSPQQHLLKSETLLSHEEDQLISNLTDSSVVSHSELFSDLFFPSDSNNSLLSPTTSGYPDNPAEDLTSSIENLTKLTCLRDKRLSSIPEQQLSSEQEQQLCLLSLRSSSDPAIALHAQQQQQQQQQQQQQQQQHQQQQQHLQLQLISPIGGPLSCGSSLPSFQETYSLKYNSSSGSSPQQASSSSTAAPTPTDQVLTLKMDEDCFPPLSGGWSASPPAPSQLQQLHTLQSQAQMSHPNSSNNSSNNAGNSHNNSGGYNYHGHFNAINASANLSPSSSASSLYEYNGVSAADNFYGQQQQQQQQSYQQHNYNSHNGERYSLPTFPTISELAAATAAVEAAAAATVSSPSVGGPPPVRRASLPVQRTVSPAGSTAQSPKLAKITLNQRHSHAHAHALQLNSAPNSAASSPASADLQAGRLLQAPSQLCAVCGDTAACQHYGVRTCEGCKGFFKRTVQKGSKYVCLADKNCPVDKRRRNRCQFCRFQKCLVVGMVKEVVRTDSLKGRRGRLPSKPKSPQESPPSPPISLITALVRSHVDTTPDPSCLDYSHYEEQSMSEADKVQQFYQLLTSSVDVIKQFAEKIPGYFDLLPEDQELLFQSASLELFVLRLAYRARIDDTKLIFCNGTVLHRTQCLRSFGEWLNDIMEFSRSLHNLEIDISAFACLCALTLITERHGLREPKKVEQLQMKIIGSLRDHVTYNAEAQKKQHYFSRLLGKLPELRSLSVQGLQRIFYLKLEDLVPAPALIENMFVTTLPF

>DmFTZ-F1—CG4059

MDTFNVPMLAESSNTNYATEATSNHHHLQHQHQQQHSHQQQQQQQLLMPHHHKDQMLAAGSSPMLPFYSHLQLQQKDATATIGPAAAAAAVEAATTSANADNFSSLQTIDASQLDGGISLSGLCDRFFVASPNPHSNSNMTLMGTATAATTTTTNNNNNNNTNNNNNNNVEAKTVRPSNGNSVIIESVTMPSFANILFPTHRSANECIDPALLQKNPQNPNGNNSSIIVPPVEYHQLKPLEVNSSTSVSTSNFLSSTTAQLLDFEVQVGKDDGHISTTTTTGPGSGSASGSGSGSGSGSGSIARTIGTATPTTTTSMSNTANPTRSSLHSIEELAASSCAPRAASPNSNHTSSASTTPQQQQQQQHHMQSGNHSGSNLSSDDESMSEDEFGLEIDDNGGYQDTTSSHSQQSGGGGGGGGGNLLNGSSGGSSAGGGYMLLPQAASSSGNNGNPNAGHMSSGSVGNGSGGAGNGGAGGNSGPGNPMGGTSATPGHGGEVIDFKHLFEELCPVCGDKVSGYHYGLLTCESCKGFFKRTVQNKKVYTCVAERSCHIDKTQRKRCPYCRFQKCLEVGMKLEAVRADRMRGGRNKFGPMYKRDRARKLQVMRQRQLALQALRNSMGPDIKPTPISPGYQQAYPNMNIKQEIQIPQVSSLTQSPDSSPSPIAIALGQVNASTGGVIATPMNAGTGGSGGGGLNGPSSVGNGNSSNGSSNGNNNSSTGNGTSGGGGGNNAGGGGGGTNSNDGLHRNGGNGNSSCHEAGIGSLQNTADSKLCFDSGTHPSSTADALIEPLRVSPMIREFVQSIDDREWQTQLFALLQKQTYNQVEVDLFELMCKVLDQNLFSQVDWARNTVFFKDLKVDDQMKLLQHSWSDMLVLDHLHHRIHNGLPDETQLNNGQVFNLMSLGLLGVPQLGDYFNELQNKLQDLKFDMGDYVCMKFLILLNPSVRGIVNRKTVSEGHDNVQAALLDYTLTCYPSVNDKFRGLVNILPEIHAMAVRGEDHLYTKHCAGSAPTQTLLMEMLHAKRKG

>DmHR39—CG8676

MPNMSSIKAEQQSGPLGGSSGYQVPVNMCTTTVANTTTTLGSSAGGATGSRHNVSVTNIKCELDELPSPNGNMVPVIANYVHGSLRIPLSGHSNHRESDSEEELASIENLKVRRRTAADKNGPRPMSWEGELSDTEVNGGEELMEMEPTIKSEVVPAVAPPQPVCALQPIKTELENIAGEMQIQEKCYPQSNTQHHAATKLKVAPTQSDPINLKFEPPLGDNSPLLAARSKSSSGGHLPLPTNPSPDSAIHSVYTHSSPSQSPLTSRHAPYTPSLSRNNSDASHSSCYSYSSEFSPTHSPIQARHAPPAGTLYGNHHGIYRQMKVEASSTVPSSGQEAQNLSMDSASSNLDTVGLGSSHPASPAGISRQQLINSPCPICGDKISGFHYGIFSCESCKGFFKRTVQNRKNYVCVRGGPCQVSISTRKKCPACRFEKCLQKGMKLEAIREDRTRGGRSTYQCSYTLPNSMLSPLLSPDQAAAAAAAAAVASQQQPHQRLHQLNGFGGVPIPCSTSLPASPSLAGTSVKSEEMAETGKQSLRTGSVPPLLQEIMDVEHLWQYTDAELARINQPLSAFASGSSSSSSSSGTSSGAHAQLTNPLLASAGLSSNGENANPDLIAHLCNVADHRLYKIVKWCKSLPLFKNISIDDQICLLINSWCELLLFSCCFRSIDTPGEIKMSQGRKITLSQAKSNGLQTCIERMLNLTDHLRRLRVDRYEYVAMKVIVLLQSDTTELQEAVKVRECQEKALQSLQAYTLAHYPDTPSKFGELLLRIPDLQRTCQLGKEMLTIKTRDGADFNLLMELLRGEH

>DmHR4—CG43934

MPICRIMTLSRGPYSELDKMSLFQDLKLKRRKIDSRCSSDGESVADTSTSSPDLLAPMSPKMCDTGGAGAGGGGGHGGGVGGALSSLPLPTAVVTLTATSTIAPVSAAAATICSSSTVPTAASSNTGHNVCVATPPTAATTVVVSAGKAAAIKAEQKNQQQQQQQQQQQTEINSSSGPVSAAAASSVMSPPPTTTVRMSPAPPTDLSLPAGRRTPSNATPTRATPTPPHNNGNASSSCGTSNGGGSSRASPDSMDERPSTTTTTTTGGTQMSTNGIHGGASGDCMQNAASAECGVSNLSVIRSVKEERIAASPTKMLSYHQHQQQQQQQMQYQQHAPSPTQSKGLTQSQQHVSVLVTPSRIKSELSPQQQQHAQQMPPTSLQHLAHHPAYSTSVSTATSSVTSTSGALTTTTSSSAAALHASTPSPTPLPTPAAVAAAAVAAAAAHHPHLHHPPPPIVQTHHLHQQLTQPQLRKSSPPTGLGLPQQHLLNQSMQHLTQQQQLQLLQQAAAMSQRPTQMSPNAINSSPSARHQSSASSPHQHLLQQHHHHQQQQQQQQQQQHLRIKKEPAQLLAAAAGSLLGSSGGAQRHLHSPHHHHPSSPQQQQQQPPPQLPPQALGNIAQLIHPGSLQLRHPNRDAAILFRVKNDVHQQVAAAQLMQPGAAAAAAAAAQRMVWVSNARINGVKPEVIGGPLGGLRPGGPAQSPSPHHSSSSSSSQLSPQTPSQTPPRGTPTVIMGESCGVRTMVWGYEPAPPTAGPSPTHGGNNAASTPQHPHGQQQQQPPSSMSSLSQQSHHSSQSSLQSASSPSAGSITPTHTPGPSPQNNEEAAQLLLSLGQTRIQDVRPRPHQFRTPHALNMERLWAGDYSQLPPGQIHALNLSAQQQWGSTNATGMNRGLDAPHEPTDEDDQPLVCMICEDKATGLHYGIITCEGCKGFFKRTVQNRRVYTCVADGTCEITKAQRNRCQYCRFKKCIEQGMVLQAVREDRMPGGRNSGAVYNLYKVKYKKHKKSKQQQQQQQQSQQQHMHSPHHQSPLSPHHQQHLLSPQQHLHQHQQQLAAAAAVAAAAQHQHSKLLSATLGQSEMKPMFLGPSIKQELLQQQQQQHQQQQAAQLHSPHQQQLHSPHHTLHGSPHGALPPHSSASALHSPLSHGPPSLPPHTSSSSSSSSSASSGGSSSSSQQPSHHALHSPHHALAHAHHLQANASLAPPHAQQLHQQQQQQQQQQLHQQHASQLQENAIKLPSHLVNGTILKTALTNPSEIVHLRNRLDTAVSSSKDRQLSYEHAHSMIQTLIDCDAMEDIATLPHFSEFLEDKSEISEKLCNIGDSIVHKLVSWTKKLPFYLEIPVDIHTKLLTDKWHEILILTTAAYQALHGRRRTSSGGRSSAGAPPHSPASANHHHNHHHMSGSPHATSLNLPHQLHHQQMQAAATPPLQKEDPEFVDEVNGHLLTLQTCLTTLMGQPIAMEQLKLDVGHMVDKMTQITIMFRRIKLKMEEYVCLKVYILLNKEVELETIQERYVQVLRTYLQHSSPQNPQDRLTELLSHIPEIQAAASLLLESKMFYVPFVLNSASVR

***Bactrocera dorsalis (Bd)***

>BdKNRL—XP_019844421

MNQTCKVCGEPAAGFHFGAFTCEGCKSFFGRSYNNLSSISDCKNNGECIINKKNRTACKACRLRKCLLVGMSKSGSRYGRRSNWFKIHCLLQEQQQQAAAAMAAHHNNQQQAAVAAAHAAAQHGGPPLMPGQKPPVPHGLSPHGPPSSAAAAAALGMLGHAGAYPGLYPPRTKEELLMLGLEEYAAAAAAAKHPATASPAVSSPDSHNSDNSLEMSTRGSAGANALLHLSAKHAPEAGGASATAPPPPQLRKDLPHFLPLPFPGLGSMPVMPPPAFLPPSHLLFPGYHPALYSHHQGLLKTSAEQQQQAAAAAAAVHQLFNSNNVTQNRLPPAMQAPSSPLTTATTAPYSNKLDAFKDAERVTQSSKCGDALNNNHIPNGSKAAEELTKRFYLDAVLKSQRNSPPPTTTKLPEHPKHDYSISALVTPNSESGRERLRSRQSNDEDEECEERNDITRRSTTAANDGHALIGNGGDTDERAKAQCDNDEDDDEELVVSMTPPHSPVNNRTLNSGADNGNTVTNTNNTTNNNTTPATTPTPAVATLPTVSPTAAKSSHQQDNPIDLSMKTCSSVASSSLSSKCSSTGSVQATDSDNEIECVGEQEEQTKCRQHGKDAEKQMQSFKPLAECAVEPLLQVDIDDSDKPIDTRSLKLCDSNKNNNNNDNNSANNNNLINSSDSDESVLQLDTVKRKLQPVGSKPSEADVLSAKRLKLSAAATALECVANIKSGVDDCVGAASKATTTTPLDLTTKV

>BdEGON—XP_011213892

MNQLCKVCGEPAAGFHFGAFTCEGCKSFFGRTYNNISAIAGCKNNGDCVINKKNRTACKACRLRKCLLVGMSKSGSRYGRRSNWFKIHCLLQEQQSVPGNGPGGGGGPGGGGNISDNNANRASFEQLARLHQLQARQTYQDKTNPCIKSASMSPTALALSAAAAAAVAANSRGSPQPPPAQHNLLSSAAKFLNGVADSGRHHAHQQQQLQQQQQQMAQQQLQHSAQQHQHLHHLPAFGYGGKAETRLSETPSDSGASSAGDAADDARSSVSGRQERNCNSAQHLAALTYDDLEEAEARERRHQQLLQIYRSHSEPLRSPFAHFALPPHISPLAAPPGTLLSTTNIAGSPPAHLMPAALKEEIKRLSEAASAAAAAAAASAASAAVADGASNNDAQEEPIDLSLKGRAIERRWAAARSSYERQLAHGEGNSLTSTTPPPSSASPTALSTAAHAALAHAVIHKPTPLDLTSVRSQTLTG

>BdKNI—XP_029404449

MNQTCKVCGEPAAGFHFGAFTCEGCKSFFGRSYNNLSSISECKNNGKCVIDKKNRTTCKACRLRKCYTVGMSKGGSRYGRRSNWFKIHCLLQEQQQQAMDIANKAAAAGGNVAGAPSAAGFGDMAAAAAAAAAAKQHMQSLNPHMASAGGLLGYPGYQFPEMMAQAAPFMSAAARHPAADATFFNMMNSLPAMGAAQSAFQLPPHLLFPAAAAAAAYHPNAAAAAAAAADAAYRTEMYKHRQSVDSAASGDSAQRFSPQIAAAAAAAAVEQQMREQQEAARHSPELCVSGGDEDEDDELDVHTPSSAIAQQQIYQQMQEQQHQQQHQIHSPVAIHASPLSNSPLCVPVPTSALTPNSEHSPAHQPSSPAPTVAETAALSFAAKMQSLSPVSVCSIGAENVSPQQQHSAMDAQDGPMDLSMKPVSAASRPSTRHSGDCDMNCDLSEEEAAMLDTRRKYFVYNSDHSESDTSSDGSSAAALANKRQKMIDEGYNSAGYASSHGSTVSTSSATSMHGIFVCV

>BdE75—XP_019846264

MVCATQEVNGNERQQQHQRQPIQLQHLPETSQPRQLKSLAIQSTENQFPKIVLLSTSATNINQNNVTQTTSSRQLKQQHSALVKLLESEPITKKIVTKNTDTNIGNADLLIKHELNVKPNDQDCLNLKKKNEQKQQQFIHNPSATQCKRLKSNGINHPLLVEHTTTKNHSVKTGTTCSFTINAIYETKTQDRVDDNENNFVNKLKCRDLGDDDHDNKFKERIANTPHHSKLSIPQTDVATLHSSNDFFEQCQCPWKKIRFAREWKQREQLQKRKQPELQSVEGSKQILTDEKLESNDGNKEGDNTILNSKGIVATPEFVLSKQTEIEITTLLKQQQQFRRDSSDSNSSLLSNSSTQSTTCVNFCTCTPQQLKYQQDSVNGNDTSSSFCQFSDNDNTEIGCDDELCMVHNTSHQQSQLEQSAMNDLCQQFDDNLETFTNFTPEVDSKSVLNNDLLTSGVKNILHNPSSYKIDAETGVPDGCKGFFRRSIQQKIQYRPCTKNQQCSILRINRNRCQYCRLKKCIAVGMSRDAVRFGRVPKREKARVLAAMQQSTQNRGQQRALAGELDDQPRLIAAVLRAHLETCEFTKEKVSAMRQRARDCPSYSMPTLLACPLNPAPELQSEQEFSQRFAHVTRGVIDFAGMIPGFQLLTQDDKFTLLKAGPFDALFVRLICMFDSSINSIICLNGLVMRRDAIQNGANARFLVDSTFNFAERMNSMNLSDAEIGLFCAIVLITPDRPGLRNIELIEKMYSRLKGCLQKVISQNRADQPDFMSKLLDTMPDLRTLSTLHTEKLVVFRTEHKELLRQQMWTMDDEQSTNIKSPVINWDDARMDVEAKSPLGSVSSTESVDLEYSTSSTTSAPHHRVNNLDQQPSALASSTPLLAATLSGPCPLRNRANSGSSGDSTTEMDIMGSHAHLTQNGLTITPIVRSHSHQQLHHHLTTGAPNRYRKLDSPTDSGIESGNEKNDCVIKAVSSGGSSSCSSPRSSVDDALDCTDTPAASTSQVKLSVSPVRSPKPIAPTSTSSLTGIVSTPNSLKRQIVEDMPVLKRVLEAPPLYDTNSLMDEAYKPHKKFRALRHREIEIAEADPSSTSNNSLNTEKITTPKADANNTVQSSQVATAASSPPPFIQSTGTACTPFVTSHCTVTTASSPHSHAITGSSLNSHHQSQLHMHLTRHTSSNHNTSSNGNQNHTSNVPPQQQSSLSSTHSVLAKSLMAEPRMTPEQIKRSDIIQNYIMRDQASCSSGSLIPGTPTHTRTRSPAPVQHLSAMNQTSTIIHLLLPHLQVVLQLAQAQSAVHLRLQQ

>BdE78—XP_019846486

MCSVASSPSELGCTGQSSVANTANTPGPGGVGGMGVGVNMSVSVGAGGAGGMMGQCGNVSVGVGGMSVVGNGGSVGGGGGGVGGNVGITSVDNNGCGVVDMQGPTAELTVYDVIMCVSQAHRMHCSYTEEQTRELMRRPVAVPQNGIATSVSETMEFQKIWLWQQYAGRVTPGVQRIVEFAKRVPGFCDFTQDDQLILIKLGFFEVWLSHVARMINDATLTFDDGTYLTRQQLEILYDNDFVISLVNFANTLNSYGLSDTEIGLFSAMVLLASDRSGLAEPKMISRLRERVAEALRVQLVRSRAGSTQALQLMPALEAKIPELRSLGAKHFSHLDWLRMNWTKLRLPPLFAEIFDIPKTEDDL

>BdHR3—XP_011208441

MYTQRMFDMWSSVTCKLEAHANNLGQSNVQSPGHNNSSGSIKAQIEIIPCKVCGDKSSGVHYGVITCEGCKGFFRRSQSSVVNYQCPRNKQCVVDRVNRNRCQYCRLQKCLQLGMSRDAVKFGRMSKKQREKVEEEVKFHKAQLRAQSDAAPDSSVFDTQTPSSSDQLHHNYNGYGGGYSSNEVSSPYGYGYSTSVTPQQTMAYDISADYVDSTTYEPRGTIMDSDFVSHADGDINDVLIKTLAEAHANTSTKLEAVHDMFRKSQDISRILYYKNLGQEELWLDCAEKLTQMIQNIIEFAKLIPGFMRLSQDDQILLLKTGSFELAIVRMSRLLDLSQNAVLYGDVMLSQEAFYTSDSDEMRLVSRIFQTAKSIAELKLTETELALYQSLVLLWPERNGVRGNTEIQRLFNLSMNAIRQELEANHAPLKGDVTVLDTLLNNIPNFRDISIMHMEALSKFKQQHPNVVFPALYKELFSIDSPQDLT

>BdECR—XP_011203444

MLEGRDDLSPSSSLNGYSANESCDVKKIKKGPAPRLQEELCLVCGDRASGYHYNALTCEGCKGFFRRSVTKNAVYCCKFGRSCEMDMYMRRKCQECRMKKCLAVGMRPECVVPENQCAMKRREKKAQKEKEKQTTGNSPIICKSESIKNEILELMNCEPPSHPTCPLLPDDIVAKCRASNIPPLTRNQLAVIYKLIWYQDGYEQPSDEDLKRITSTPDENESPNDVSFRHITEITILTVQLIVEFAKGLPAFTKIPQEDQITLLKACSSEVMMLRMARRYDHNSDSIFFANNRSYTRDAYKMAGVADNIEDLLHFCRQMYSMKVDNVEYALLTAIVIFSDRPGLEKAELVEAIQNYYINTLRVYIINRHCGDTKSLVFFAKLLSILTELRTLGNQNAEMCFSLKLKNRKLPKFLEEIWDVHAVPPSVQSHIQAMQAEQESLEANAAAVATTSTSAAASSSASGSPTDMIGVGFANNSPNSTSVSTSAIGMNGGLSSATTAGTSSLYGMPFKQEYIMGPGSGIGIGGNLTLKSEHLIGMQLSNDHHMMGIQLKQEHMMGGVDHNTAA

>BdHR96—XP_019847193

MLCTKDSVEMSPPKSCAVCGDKALGYNFNAVTCESCKAFFRRNALAKKQFTCPFSQNCEITVVTRRFCQRCRLKKCLDIGMKSENIMSEEDKLIKRRKIENNRAKRRLNDNKAGSITDSTAFDMVSHDSQSSFDSGKGTGSNNSASAALGNQSSPLSANVAIRSPIRSPMLTESDGEAKPELMTSEEIVEFIVGDPDRASQMISKLMRTKDEALAIVEKILTSQKDALRLVSHLIAFPGDALKIISKIMNSPFDALTVFTKFMSSPTDALEIISKIVSSPQDVVQFIRNLMECPEDALDIMNKFMNTPAEALRIINKIFNTLNPPTRSDELHKLLLDATKENKDKTFQPTPEASTESEGTENGNYLLHSMLNNSPVMTHDFVNGVSPRDQRSMSVTNSDIMVESPINIQGMTIRTDTPDHTLTPIQSQTLQLGSSAELPSVTACMENSTQGISITPTDCSIDNHALHCNSPTSIQSNIELPSTSAGLLNPNSPTDLTSDATDFNSEHFDIKTFIQNSFPDNASGGDSLNSLESVLSEVIRIEFQAFNNLPPEPRVKQEQFQYQTHNMAMQNSVQQQQCGYNGGHTQLQQPIIGPPTNAIMGRDLNEAEHMKLRELKLASEALYYPMDNDLSLLMMGDDRVKPEDTHQDPKLLHVINLTAVAIKRLIKMAKKISVFRDMCQEDQVALLKGGCTEMMIMRSVLTYDNNRNTWKLPHVSNMAHVRAEILKQAKGNIYEEILKFVGTFDEKWRMDENIILIMCAIVLFTPTRARVIHADVIRLEQNSYYYLLRRYLESVYPGCEAKSAFIKLIQKISDVERLNQFVIGVYLNVNPSEVEPLLREIFDLKNH

>BdHNF4—XP_029404261

MLKMKTEVLSGSGFDESYIFEDNLLRMIDAEGHMMHGIEPALSALSSASAHSPSGSQALSPALSLSGGNSNSNGNLSTSGNNTINNNNNSYAQNNNSQSATVCAICGDRATGKHYGASSCDGCKGFFRRSVRKNHQYTCRFSRNCVVDKDKRNQCRYCRLRKCFKAGMKKEAVQNERDRISCRRTSNEDPDPGNGLSVISLVKAEIESRQSKAGAAMETNPNEDLSNKQFASINDVCESMKQQLLTLVEWAKHIPAFNDLQLDDQVALLRAHAGEHLLLGLSRRSMHLKDVLLLGNNCVITKHCPDARLSPNLDISRIGARIIDELVFALRDVNIDDTELACIKALVFFDPNAKDLKEPQRVKTLRHQILNNLEDYVSDRQYESRGRFGEILLILPVLQSITWQMIEQIQFAKIFGVAHIDSLLQEMLLGGELADNSPLSPPSLTNYSPTRNMDGSHVSSTLDVLTSPTTADHLSACMDSNSLDAQMMSPLLENIAASPPQYNQLRSYQTQQRPQQQQAVNNNTGTPLHTRTMHSPHLNDADANNVDMVNGAGNSCMDETGMYNTNSVMRPLSTSSAHNLPHMSSPNMQQHPHAASSPMQHSPPLHRNHPYQRPDQLNAAQQHNNGAVAQQLRNPAEMTLNEYNSGSAEEMLRRAPIKIRGPDALSSGAGGAYMMGGGLHDAESAYRLTLKQEPETGY

>BdUSP—XP_019845796

MDGGEQEQSFRLGPLSPQEVKPDISILNENNTGGYSPKSASPVSFGVIGLQSMNMTNAGSVAATVASHNPNQIQHGTQQQQQQYPPNHPLSGSKHLCSICGDRASGKHYGVYSCEGCKGFFKRTVRKDLTYACREDRNCIIDKRQRNRCQYCRYQKCLACGMKREAVQEERQRGTRSQNVRSSDDFNPSSSVRDLTIERIIEAEQKSEQLSGDNAIPFLRVGPNSMVQPDYKGAVSHLCQMVNKQLYQMVEYARKMPHFAELQREDQVLLLKAGWNELLLANVAWCSIDSLDSDYATSGGHDTSSGRRSPIRQPQQLFLNHNFSYHRNSAIKAGVATIFDRILSELSVKMKRLNIDRSELSCLKAIILFNPDLRGLKGRNEIEVCREQVYACLDEHCRTEHPGDDSRFAQLLLRLPALRSISLKCLDHLFFFRIMGDKQLDQLFYEQLDAPMC

>BdHR78—XP_011201915

MGASGFKNEDNKTAATIVHHVTGSGNGTSGGGVTNATTATNMSIETCLVCGDRASGRHYGAISCEGCKGFFKRSIRKQLGYQCRGSMNCEVTKHHRNRCQFCRLQKCLASGMRSDSVQHERKPIVDKKEFAGSSSSGSSNSTVTNAAAAAAVLGANNAATGSGSLFFGHNLTTNAATVNKSEAPTTSKPSIFSYNMTLPEMRQAFVNNTDVSVPPLPFHLTFAEMRQIIENAKNFQQEKQVPIQNPPPQSLYFSPDLPKADDEEDDDDESMDNSSTTCLQNLTTNANNNNTQNLNLNIDAIHSPATAVGQIQTALDRCVIEKALQLLIPIQNQLDRLSLAAGNGNAGSSPAIKNEITDDDMGEDSSADDEYEVAETILGVANTKEFVANECIFDNEILTSAQAAFNLQTPTLMSAYLNLHYICETGSRVIFLSIYWMRKIAAFDQLDGRTQIKLLRASWPGLLAIALAQVRSLSITTIITTLVANVRQLAEVDKIEPQKIRKLSEHIARIHSYIQETLALDLDDMEFAFLRLAIFFNPHMLLRKRERNVRDFVRRVQLYVLSSFRKHIASQLDDEQQAEERFSTLIITLLPLAALESDVIEELFFSNLIGQVQIDNMIPYILTLSANTSL

>BdSVP—XP_029407513

MITHNSLSVYGGGVMCASPSTAPGFFNPRPQSGAELSAFDLGLPRSMALGVPPPSAWHDPNLGGHLHASAGPGSLAGTAGAGGSSVSTGGGTTPSSVASQQSAGIKQDISGLTGAGGQSNTHNQSGHHAIKEDLSSLTAASAAASAAAHHAAAVHHAAAAQQNHGQELGVMIKGQQTGSSCLGASSTAGGGGNSGGGGGSTTPSSQANSLHSQSSNSDSKQNMDCKQNIECVVCGDKSSGKHYGQFTCEGCKSFFKRSVRRNLTYSCRGSRNCPIDQHHRNQCQYCRLRKCLKMGMRREAVQRGRVPPTQPGLAGMHGQYQLANGDPMSVAGFNGHSYLSSYISLLLRAEPYPTSRYGQCMQPNNIMGIDNICELAARLLFSAVEWAKNIPFFPELQVTDQVALLRLVWSELFVLNASQCSMPLHVAPLLAAAGLHASPMAADRVVAFMDHIRIFQEQVEKLKALHVDSAEYSCLKAIVLFTTDACGLSDVTHIESLQEKSQCALEEYCRTQYPSQPTRFGKLLLRLPSLRTVSSQVIEQLFFVRLVGKTPIETLIRDMLLSGNSFSWPYLPSM

>BdTLL—XP_011214380

MSSREVSPDPAEKYNRLSPSASSRILYHVPCKVCRDHSSGKHYGIYACDGCAGFFKRSIRRSRQYVCKSQKQGICVVDKTHRNQCRACRLRKCFEVGMNRDAVQHERGPRNSTIRRQIQIYKEVVMGPDPLPSELMMSHRLFNAFPMSPMLIPTVPRPPPPPPPPPQHGRPFPQPPPPPSQQYLPPTQQTGPVTFQQQSSASSSSNFENSYSSSQHNEQFIHAQALPLQQEPRFTQVHDCGQGPNLVSAGYQLQQQNQYQQQNQYQQQQQQQFQQQHSQQYSASNSVSSTSANAYNAISQSIPVAETHTQFVEQEPADSYGLPSGGNHLDHDYDGYASQKSSVAALPDGTNPQELPGLDGLNVISAQKSQSIQLSPEQVSGAAHSQYEPTFQLDIAGNGAKSVQTEQSANHEEILSEGLLQSILTAIEQPQQKGVQHQHTVQVITNQNNDIYGHGAQSRSDTDIQEDATPDDEHEPEVAESDKVEVQVNADGEEVATVQEIKPIVAAEIDDDEAKH

>BdHR51—XP_011200189

MVQNERQPRNTATIRPETLREMEHGRALREAAVAVGVFGPPVLLSPPCYGPGLLPPPSLSNLPTNRRLHHNHISKALQLSSNVSQAGGFSVFNNNIQFSKDNSYATSMDLSNGTVSVSTNSSSTNSIDPSSPISDTVLDKNPVGGSVSSISSTSSVHDNDNDDDSIDVTNDEEPLRNEKLDCHFTIPTFVPQNLYVQPHETVYETSARLLFMAVKWAKNLPSFASLSFRDQVILLEESWSDLFLLNAIQWCIPLDPTGCTLFSVSEHCNNYDTTQESENCVTKEEVAASVRTLHEIFYKYKAVLVDPAEFACMKAIVLFRPETRGLKDPAQIENLQDQAH

>BdERR—XP_019846473

MSEGGVLHIKQELDISTSCCSPSTSTTVGVNRSNSYSNGTSGSTSSTSSGSAISGGGTGGNTSTSGNKSSPSVSPERQLCSSTTTLSADLHSVTLTGCEASTPIALKSNDCGGNNTNSNNSAGSASGGSIRDELRRLCLVCGDVASGFHYGVASCEACKAFFKRTIQGNIEYTCPANNECEINKRRRKACQACRFQKCLLMGMLKEGVRLDRVRGGRQKYRRNPISNTYQTMQLLYQSNSTSLEDIKILEILNAYEPDTLTVQQQQQPHTTTESSNNTGTGKATQNTSVKLEQNNCSSGGSNSNNSEILSSSAQPICIFQNNNCDAEEILAVLSDIYDKELVSVIGWAKQLPGFTELPLNDQMKLLQVSWAEILTLQLSFRSLPFNGRLCFAADVWMDELLAKECGYAEFYYHCVQIAQRLERLSPRREEYYLLKALILSNCDILLDDQSSLRAFRETILNSLNDVVCLLRYGSAVSHQQQLLLLLPSLRQADGILRRFWRDICHEGKITVKKLFVEMLEPVSR

>BdHR38—XP_011212592

MSQSTHIIGRVRSERFLATKPDEDRRRRTIIIEKKNDSYGFTLQSYGIHYKKDQEVEMITYVDYVEYDGPAYKAGMREGDVILSINGSDMEKADHKTIVDFIKSCDTRMRMVVLFEDCVRKVDLHMRYIQLQNLLHSKMNELERICLRERELLEGKWKTHSLPARKKATTSPTDAESGTSPTDGENLPYYRPALSTEDVPSMARFQSQQQAIIPPPAQFTLTYHYLDPTYRYVMKPVTSNSSGEHLATSSCGIEGIQRSPSNQHFILRHTESVDTATGLASLSHRSVLSQAQSGASAQAPVPPPRTCEKHKPPNKQHSVEQQHYQQQQQQQQHQQTTTPVMSQKAHKSSKHCYGHSCNPCIGHFRWKSAEKAAATAAATMGGAGGMNVAGGNGNGNGDNISLDAYDLASPCCDAHCVPTRRRTRHKEHTHKHKHRDRERDSKERQPRPKSQTSHTSPGITRHHMHHHHHHTAQDMLQQQPQPQQQQQQQQQHHHHHHLSPQQQQQQQHQHLQQSQPQPQPHGNCDSHNGSMRSRYYDLTTGLMSHCSLHSCTSSEFAPADSTSYTTSLSTDTLYWDPKSEHSASRQHSTKSRQSYQQYPQQHAQQSQQQPHTQHHQQQQQPTAHVHGVYQQRYHITATQVQPSQIYPQATYVQKPKSWDNLTTKAVSGYGFGYGYLDTVTVKPAMKLQIAQQRHSIPRKNPYGRYSTYTDVENYAPPPSQFVEELITTTTTTKIMAKSTEELIAAPVPQTTCDCMTKQQQQALKAAQYQINQAAKVSHHNNCQMGYYSHLPRPTLDAGTSTVQTTTTGVSGDVATVSEVTRL

>BdFTZ-F1—XP_029405285

MLLEMEQQQATASFISSLNMSPFTMQLDPQQPSSPALGGAGGPNNGQTNASSGAAANRSNTDANALSGGDADYSSYALHLSAGGGGNAGGGNSDGSGATNAGSTHHHHHHQQQQQQHHQQQQQQQHYQTQQQPQQLQQQQQQNIANNPNFSYSYIYNFDSQYVFPTGYQDNTSSHSQQSVSGGGGGAGGGGGGGSSTSSNATNVVLLNGSNANAAGGYMTLLPPATSSGHMGMSSGGTGASAGGSGEGIDFKHLFEELCPVCGDKVSGYHYGLLTCESCKGFFKRTVQNKKVYTCVAERSCHIDKTQRKRCPYCRFQKCLEVGMKLEAVRADRMRGGRNKFGPMYKRDRARKLQVMRQRQLALQALRTSIGSVEMKSSPLSPGYQQPYPNMNIKQEIQIPQVSSLTQSPDSSPSPIAIALGQVNTSGGVISTPMNGGNGSGGGGGGGAGGGGGSGNTSTNSNNSNNTSSDGLNRSGSGNCHDTGTGSLQNASESKMCFDSGTHPSSTADSIIEPLRVSPMIRDFVQSIDDREWQTQLFALLQKQTYNQVEVDLFELMCKVLDQNLFSQVDWARNTVFFKDLKVDDQMKLLQHSWSDMLVLDHLHQRIHNGLPDETQLNNGQVFNLLWLGLLGVPQLADYFNELQNRLQDLKFDMGDYVCMKFLILLNPDVRGIVNKKTVLEGQENVQAALLDYTLTCYPSVTDKFRRLLSILPDIHAMASRGEDHLYSKHCAGSAPTQTLLMEMLHAKRKV

>BdHR39—XP_019844854

MSSTKAESAAGANPAVAAFGSTLAGSAFQLPVNMHAQLAANGSNGHSTNGTSTTTTTTTTRNNISVTNIKCEMDEGVVATLMPNGNNIVPVIATTGAASYYNGASVGGGGGSGVGIRIPVTKAEDSDSEAELTNIENLKVTRRAHNNADSSSGTMGPGGTVSIGGGDSANNGNHNKNGPRPMSWEGELSETETTETELMETENSLSNSGVATHNFNATSIGVAVIKSEATTTTPGHTLAPTPTHPIKPEQMEQISVEVHGGALAGLGPTMRSGLGSSSSAGSLYSATHTKLAPTQSDPINLKYEPDAAATAAGLMPTGANVNSPLLTRNKSATLPLPANPSPDSAIHSVYTHSSPSQSPLTSRHAPYTPSLSRNNSDASHSSCYSYSSEFSPTHSPIQGRHAPPAPALYGNFNGSLHHSVIYRPISVADGAAGGVTGNGNAGGNCNTEAQNLSMDTKVSQLNIGLEGLPASPAGISRQQLINSPCPICGDKISGFHYGIFSCESCKGFFKRTVQNRKNYVCVRGGPCPVSISTRKKCPACRFEKCLQKGMKLEAIREDRTRGGRSTYQCSYTLPNSMLSPLMCPEQQGVAAAVAAAAAAAANQQQQQHGRLGAPMAHSMSAQQQQQHTLNGLSAGLGAGQSIIKSEQCDDSSASARATNIPTLLQEIMDVEHLWQYNEAELARLNQPISTSSSSSSNASSSSSSTASAGGTATGNPQMTNPLLASAGFGNGENTNPDLIAHLCNVADHRLYKIVKWCKSLPLFKHISIDDQICLLINSWCELLLFSCCYRSIDTPGEIKMSQGKKISLAQAKSSGLQACIERMLNMTENLGRLRVDRYEYVAMKVIVLLQSGKCYV

>BdHR4—XP_019846713

NANQLASEVAARTANTIGNTNNNSSSSSNTVNDTGNTDVANESMNLSVLGGKHEKIAKHLKSVAASKADVNDEGSNCGAAGGNNNNGKSGSYEPKQNGQYRESDGRLDGRETPINSWQPQQREQSKQQQQPQHHRVTGEKSATAPGHNSNSSNSNSSSGSGNGNGSASKRESNADTPAARKQRHEEDADEAAHESARERVASNNDEGDFSTEHLHTATIIKTPLNKTLLKRCSSYSALGCGSNSGSGNGSGSNGNGSGNGQGYGNANTAAASAYDNAPCCARSAGNETALNCSGRDEREREREQDKQQANAEQASFNGGDECGSPQHSPSTSACGFAHKMPTTPATTTAGGSGEEAGDSNAAKCSKCNNNNNIHYNNYNCYYRKKSRMPICRIMTLSRGPYSELDKMSLFQDLKLKRRKIDSRCSLNSDGESVADTSTSSPDLLAPMSPKMCDTGGAGAGGGGGHGGGVGGALSSLPLPTAVVTLTATSTIAPVSAASTAATICSSSTVPTAASSNTGHNVCVATPPTAATTVVVSAGKAAAIKAEQKNQQQQQQQQQQQTEINSSSGPSLVSAAAASSVMSPPPTTTVRMSPAPPTDLSLPAGRRTPSNATPTRATPTPPHNNGNASSSCGTSNGGGSSRQQASPDSMDERPSTTTTTTTGGTQMSTNGIHGGASGDCMQNAASAECGVSNLSVIRSVKEERIAASPTKMLSQQYHQHQQQQQQQMQYQQHAPSPTQSKGLTQSQQHVSVLVTPSRIKSELSPQQQQHAQQMPPTSLQHLAHHPAAYSTSVSTATSSVTSTSGALTTTTSSSAAALHASTPSPTPLPTPAAVAAAAVAAAAAHHPHLHHPPPPIVGGQTHHLHQQLTQPQLRKSSPPTGLGLPQQHLLNQSMQHLTQQQQLQLLQQAAAMSQRPTQMSPNAINSSPSEARHQSSASSPHQHLLQQHHHHQQQQQQQQQQQHLRIKKEPAQLLAAAAGSLLGSSGGAQRHLHSPHHHHPLSSSPQQQQQQPPPQLPPQALGNIAQLIHPGSLQLRHPNRDAAILFRVKNDVHQQVAAAQLMQPGAAAAAAADMAAQRMVWVSNARINGVKPEVIGGPLGGLRPGGPAQSPSPHHSSSSSSSQLSPQTPSQTPPRGTPTVIMGESCGVRTMVWGYEPAPPTAGPSPTHGGNNAASTPQHPHGQQQQQPPSSMSSLSQQSHHSSQSSLQSASSPSAGSITPTHTPGPSPQNNEEAAQLLLSLGQTRIQDVRPRPHQFRTPHALNMERLWAGDYSQLPPGQIHALNKTLSAQQQWGSTNATGMNRGLDAPHEPTDEDDQPLVCMICEDKATGLHYGIITCEGCKGFFKRTVQNRRVYTHHCVADGTCEITKAQRNRCQYCRFKKCIEQGMVLQAVREDRMPGGRNSGAVYNLYKVKYKKHKKSKQQQQQQMKQQSQQQHMHSPHHQSPLSPHHQQHLLSPQQHLHQHQQQLAAAAAVAAAAQHQHSKLLSATLGQSEMKPMFQGLGPSIKQELLQQQQQQHQQQQAAQLHSPHQQQLHSPHHTLHGSPHGALPPHSSASALHSPLSHGPPSLPPSHTSSSSSSSSSASSGGSSSSSQQPSHHALHSPHHALAHAHHLQANASLAPPHAQQLHQQQQQQQQQQLHQVHQHASQLQENAIKLPSHLVNGTILKTALTNPSEIVHLRNRLDTAVSSSKDRQLSYEHAHSMIQTLIDCDAMHEDIATLPHFSEFLEDKSEISEKLCNIGDSIVHKLVSWTKKLPFYLEIPVDIHTKLLTDKWHEILILTTAAQPYQALHGRRRTPSTATPGGGDTTTAQASSTTSSSGGRSSAGAPPHSPASANHHHNHHHMSGSPHATSLNLPQEHQLHHQQMQAAATPPLQKEDPEFVDEVNGHLLTLQTCLTTLMGQPIAMEQLKLDVGHMVDKMTQITIMFRSIRIKLKMEEYVCLKVYILLNKAEVELETIQERYVQVLRTYLQHSSPQNPQDRLTELLSHIPEIQAAASLLLIKESKMFYVPFVLNSASVRXQRLERGLLPIDDDPPKPKRLMMIMQQQQQQQQENEEPAEPKVEQQQQASNLMLTKARRRAATEMAERLQRQQQEDFQKQQREQSQKSAAEQQQAQPVRFCLPMLELPTIKVENVQQQHSYTMDEKRDDADTDEEELRNLEQRQQQQQQQKQSGEENTSTHMKSPIMARLMEPTTSILKTSLLAGAAATLATLTAAAEQVNRKAQSPLTPGGRVGTRALTAQQTPAADNSLTESKRLQTQQQQPQHVQQLQQEQREQPKPAMPYKSILQTALMGERPPQSQTPPTPTTVGPKFMRASGLQAAAPPPPHVLKTAVTLASLSEIAAARQQQEEEERRLRTEQQQQQQQQQHLLLQQTLPLKKKKSFLQQKLTATCNMLTTAATLLQQQQQQQQQNTITILPVETSNNNNSTTHTANSEANAAAANTKFVLNNTSAGVLRCVSSATATATTTATTTRSPATFVNNLRRGASVTEIACNNASSGTCQAIATQTIKIPTVRTATVTTQTQTEEMHSAAAATSGAQTPLHTLAASSHVITRKLSTALQQQQQARATATTTSGSAATTLLSPPTQQSNVARHASPQRQTTLVTGTTTNACTISAAPPTSTATATATAAPPSTAVTVVVFKTGTLLPDDPRQRPAPQPPPPPRTPTATSASVTVVVFKTMLPDD

***Ceratitis capitata (Cc)***

>CcKNRL—XP_012155579

MMNQDNPYAMNQTCKVCGEPAAGFHFGAFTCEGCKSFFGRSYNNLSSISDCKNNGECIINKKNRTTCKACRLRKCILVGMSKSGSRYGRRSNWFKIHCLLQEQQQQAAAAMAAHHNNQQQAVAAAAAAAAAQHGGPPMMAGQKPTLGMPPHGTPGSAAAAAALGMLGHAGAYPGLYPPRSKEELMMLGIEEFAAVAAANKHAATASPSVSSPDSHNSDNSLDVSTRGNASASALLHMSAKHAVEVSGGASASTTVPPSQQVRKDLPPFLPLPFAGLGSMSVMPPPPFLPPSHLLFPGYHPSLYTHHQGLLKTAAEHHQAAAAAAAVHQLFNANNVAQARLPTTLQAPSPPLSANNSNKLDVCKDTEQVMPRPKSGDALNNNHITNGSKAAEELTKRFYLDAVLKSQRNSPPAIMPTTATTTTTTKLPEHTKIDYSISALVTPNSESGAEHRINRQSNDDEDERESNARRTTATKSTPPNTADDIRSCAGNGGGDVSRTTKASSDADEDEELEVSMTPPSSPANLRATSFESNTKTANPTITSTPATLPMPAIASPATSTPPVVTTAKSNLQQDNPMDLSMKTSSSVASSSLSSKCSSTGSVQATDSDTEIECVTEQEPGESRSFANAVDKQSEVSINEDTEEEMLQVDVEGLDKSPEKQAPQQSNNNNNNEKNSNNNNNNNSESNNHPNNNNKSNDSDNHSTQQRHDSSDSDESILQLNIVKRKLHAISGKQTENKASSAKRSKFSMESTNIQLDVGSVISAAAKPVSCTTVPGAATTTTPLDLTTKV

>CcEGON—XP_012155577

MNQLCKVCGEPAAGFHFGAFTCEGCKSFFGRTYNNIAAIAGCKHNGECVINKKNRTACKACRLRKCLLVGMSKSGSRYGRRSNWFKIHCLLQEQQSVPGNGPGGGGGPGGGINEQNTNRASLEQLARLHQLQARQTYQDKTNPCIKSASMSPTALALSAAAAAAVAANSNRCSPQTPAQHNLLSTAAKFLNGQQHQQQLLATHQQLQHGAQYQQHAQQLLPAFGVYGSKAETRLSETPSDSGASSAGDAADDAHSSISGRQERNCNSAQHLASAAFDDLQEAEARERRHQQLLQIYRSHSEPLHSPFAHFALPSHISSLGVPPTTLLSAGNPTSHHLMPAALKEGIKRVSEAAAAVAAAAIASVGNTSDSETQEEPIDLSVKGREVVQRLAAATASAEVACNDQRTVHVGSNSHTPTTPPPSTASPAPMFAKALSHAVIH

>CcKNI—XP_004522172

MMDLTDTMELGAYDVGITMMNQTCKVCGEPAAGFHFGAFTCEGCKSFFGRSYNNLSSITECKNNGKCVIDKKNRTTCKACRLRKCYAVGMSKGGSRYGRRSNWFKIHCLLQEQQQQQLDAINRSTATTTNTTNTTTGYGDMTTAAESLAKHQMQSLNPHYSALFGAGGGGGGGAVGGYSYNVNEMLMSNIFMPAPTLGHRPANPTMPFFSMLPTHPGYPMLIANHVGTAGYRAPPGYFPDMYRQQKSVDSATNDLTDGTPHREADKSISVAQKMRETRQVQPQSPELCVASGGDDDDDLEEVELNVHSPPPTAPINAQSNEPQQHQPEIFGSVSLYGQRFSASPLSLNVPTTALTPNSLESHTPETFSPTSISNAPTVATTESTALAFAEKLQSLSPVSDYSNTTESTHSHTSSSGISDAQDGPIDLSMKTISSTSSVDCEMSCDLNQTEAIRKYISYQIDSDMSSDISGNDEIAQKRSKRSDEGYSSLGYASSQGSASTGSAVSSKRGIYMCA

>CcE75—XP_020716750

MSKNFKCIDTAVDNDINQISAVANNSNKNIPATSGESYIKFIKTEPLDLEMAHVQAKDIGSTQDTNVSKESNKNETNVKRLEALSPLYQHLQIQTVPPRQPRVGLLNNINNVQKIILTPRKGHTHRLSSGESGEETDDVVEHIYSQQPIAVAQSRNTQSFSTTNTRIIQRVSQTQQADSIAGPQIIISRPFPATSLTTSLQRRHSTSPSHYQRNLSTINSNINAEFSSSVQSSPHHTLQHYSPNRSPQPSPPPLSLQHHVRVIRDGRLYEEAVARSSITSTHSASPPPLLRHHQSRSAPVSPVIPRRYSMSPHETLTLTLSPQNTFETHQGHMQVVIENNKHHLHTPDVSGQYQNHDLHSPPLPPPPPPATCLQTSIKAKNSLSSQVMVKSSLTTTSNQQPQQYVLSPSTRKYILPTTRQSNSIQTTNTLRPENTHLHHLKHQQISRQLPQHQEGESNISQSLTDSSSPSSTGSRNFRIPAVISSSSSNNKIHNDHQLHSNSDTAMRPPPPPPPKIKNATMTIPNSSNGVSSVINCSRNEEPSSSIPDLEFDGTTVLCRVCGDKASGFHYGVHSCEGCKGFFRRSIQQKIQYRPCTKNQQCSILRINRNRCQYCRLKKCIAVGMSRDAVRFGRVPKREKARILAAMQQSTQNRGQQRVLAGELDDQPRLIAAVLRAHLETCEFTKEKVATMRQRARDCPSYSMPTLLACPLNPAPELQSEQEFSQRFAHVIRGVIDFAGMIPGFQLLTQDDKFTLLKAGLFDALFVRLICMFDSSINSIICLNGQVMRRDAIQNGANARFLVDSTFNFAERMNSMNLSDAEIGLFCAIVLITPDRPGLRNIELIEKMYSRLKGCLQKVISQNRADQPDFMSKLLDTMPDLRTLSTLHTEKLVVFRTEHKELLRQQMWTMEDEQPINVKSPGINWDDARMDVEAKSPLGSVSSTESVDLEYTAPSTTSISHSHINPQPSPTSLASSAPLLAATLSGPCPLRNRANSGSSGDSTTEMDIVGSHAHLTQNGLTITPIVRSHSHQQLHHHLSSSAQNRYRKLDSPTDSGIESGNEKNDCGTKTVSSGGSSSCSSPRSSLDDVLDCTDTPTSSNSQITISVSPVRSPQLTAATSTSTSTGTANIPNALKRQIVEDMPVLKRVLQAPPLYDTNSLMDEAYKPHKKFRALRHREFESTEADPSSTSNSNNTDKITTQNTEANNPIPSSQTASVTSSPPPFVQSTSTPLVPVATSPHKVATAPSSSPHSHALTGNSANSHHQSQLHMHLTRHICNNVTGNQNNLGTMQQQQQSTLSSTHSVLAKSLMAEPRMTPEQMKRSDIIQNYIMRDQAPSSVPLMPGTPTNTGSRSPAPMQHLSSLNQSSPQYTSGASHSTLSASPTSAVGQNAIRWNSHSVITTTSANHRQQSVSPNSNGSSSSSLNGCQYFQSPHSTSASVSPPCSTSAGHSPPQLLELQVDIADSQQPLNLSKKSPSPPPTKLQALVAAATAAQRYPGVSGDVTVTPSKTQTSQTVTPVPANSTQQLQVHKVMLEA

>CcE78—XP_012158149

MPTTTTILMTIAATKAFTVAATAATAITTSATSATIASLGFATVRYGRVPKRSRELNGTPSSTDDPNSLVRVNTPNTPQTPQMCSVASSPSELGCTGQNSVANTANTPGSGGVGGMGVGVNMSVSVGAGGAGGIMGQCGNVSVGNMGVVGGNGGNVGGHTNVDNNGCGVVDLQGPTAELTVYDVIMCVSQAHRMHCSYTEEQTRELMRRPVAVPQNGIATSVSETMEFQKIWLWQQYAARVTPGVQRIVEFAKRVPGFCDFTQDDQLILIKLGFFEVWLSHVARMINDATLTFDDGTYLTRQQLEILYDNDFVISLVNFANTLNSYGLSDTEVGLFSAMVLLASDRSGLAEPKVISRSRERVAEALRVQLVRSRAGSTQALQLMPALEAKIPELRSLGAKHFSHLDWLRMNWTKLRLPPLFAEIFDIPKTEDDL

>CcHR3—XP_020717659

MYTQRMFDMWSSVTCKLEAHANNLGQSNVQSPGHNNSSGSIKAQIEIIPCKVCGDKSSGVHYGVITCEGCKGFFRRSQSSVVNYQCPRNKQCVVDRVNRNRCQYCRLQKCLQLGMSRDAVKFGRMSKKQREKVEEEVKFHKAQLRAQSDAAPDSSVFDTQTPSSSDQLHHNYNGYGYSSNDVGYGSPYGYSTSVTPQQTMAYDISADYVDSTTYEPRGTIMDPDFVSHADGDINDVLIKTLAEAHANTSTKLDAVHEMFRKSQDISRILYYKNLGQEELWLDCAEKLTQMIQNIIEFAKLIPGFMRLSQDDQILLLKTGSFELAIVRMSRLLDLSQNAVLYGDVMLPQEAFYTSDSDEMRLVSRIFQTAKSIAELKLTETELALYQSLVLLWPERNGVRGNTEIQRLFNLSMNAIRQELEANHAPLKGDVTVLDTLLNNIPNFRDISIMHMEALSKFKQQHPNVVFPALYKELFSIDSPQDLT

>CcECR—XP_023158465

MMKRRWSNNGGFALRILEESSTEVSSSSNGLVLSTTLSPSPLDSPVYGDQDLWYDAAFNGHGGGGGGGGHSVITSAHSCAALLPPQTSIIPLGGGVNNTNMNGAGNPGINFGGSGGGVNGIVGGGSLAHGSQLGNGHLGNQLNSHNNNNNNMNMHNNNINTTNMLHATLNQGLINGLMSGVLGVSGGMQHNGLNMQQHTPRSDSANSISSGRDDLSPSSSLNGYSANDSCDVKKIKKGPAPRLQEELCLVCGDRASGYHYNALTCEGCKGFFRRSVTKNAVYCCKFGRSCEMDMYMRRKCQECRMKKCLAVGMRPECVVPENQCAMKRREKKAQKEKEKQTTGNSPIICKTESIKNEILELMNCEPPSHPTCPLLPDDIVAKCKASNIPPLTRNQLAVIYKLIWYQDGYEQPSEEDLKRIMSTPDENESPNDISFRHITEITILTVQLIVEFAKGLPAFTKIPQEDQITLLKACSSEVMMLRMARRYDHNSDSIFFANNRSYTRDAYKMAGVADNIEDLLHFCRQMYSMKVDNVEYALLTAIVIFSDRPGLEKAQLVEEIQSYYIDTLRVYIINRHCGDSMSLVFFAKLLSILTELRTLGNQNAEMCFSLKLKNRKLPKFLEEIWDVHAVPPSVQSHIQATQAEREGLEPNTAVATTSTSAAASSSSGSPTDMVTGSSGGGFANHSPNSTPVSSSAIGLNGGLSSATTAGTSSLYGMPLKQEYIMGPGSGIGIGGNLTLKSEHLIGMQLSGDHHMMGIQLKQEHMMGGMDHNTAA

>CcHR96—XP_004521160

MEMSPPKSCAVCGDKALGYNFNAVTCESCKAFFRRNALAKKQFTCPFSQNCEITVVTRRFCQRCRLKKCLDIGMKSENIMSEEDKLIKRRKIENNRAKRRLNDNKKGSAAESAIFDTISHDSQSSFDSGKDTANSGSATTMNHSSPTGSNVPIRSPPLLESDSEAKPELMTSKEIVEFIVCDPDRASQAISKLMRTKDEAIAIVEKILTSQKDALRLVSHLIAFPGDALKIISKIMNSPFDALTVFTKFMSSPTDALEIISKIVSSPQDVVQFIRNLMECPEDALDIMNKFMNTPAEALRIINKIFNTMNPPTRSDELHKLLLDATKEKDTPFLTAQVSPVSEESENRNYLLQSMLDNSPAMPQEISNCLSPHDQHVMSVNSDVMVESPINIQGMSIRTDTPDHTIPSIQSQSLQLDSSTSIPTVAKCLETSRSSMPLPSRDCQITNHTLHCSTSTSVHHDIELPSTSAGLINPSSPTDITSDTTDFTSEHFDIKSFIQSSFPDNASGGGDSLNSLESVLSEVIRIEFQAFNNLPPEPRDKQEQLQYQANNLAMQNSEHQHDQQHQQSGISVCNTQAAQQPMCLPPNVSMGRDLNEAEQMKLRELKLASEALYYPMDNDISQLMMGDERVKPEEAQQDPKLLHVINLTAVAIKRLIKMAKKIGVFRDMCQEDQVALLKGGCTEMMIMRSVLTYDNNRNTWKLPHISNSAHIRAEILKEAKGNIYEEILKFVGTFDERWRMDENIILIMCAIVLFTPTRARIIHSDVIRLEQNSYYYLLRRYLESVYPGCEAKSAFIKLIQKISDVERLNQFVIGVYLNVNPSQVEPLLREIFDLKNH

>CcHNF4—XP_004519325

MKEPKEPLQEDNLLVNSKKMENLEGDSNDKDAEGHMMHGIEPSLSALSSASAHSPGGSQALSPALSGNSNSNGNLSTNGNNTASINNNNNSFAQNNNSQSSTVCAICGDRATGKHYGASSCDGCKGFFRRSVRKNHQYTCRFSRNCVVDKDKRNQCRYCRLRKCFKAGMKKEAVQNERDRISCRRTSNEDPDPGNGLSVISLVKAEIESRQSKAGAAMETNPNEDLSNKQFASINDVCESMKQQLLTLVEWAKHIPAFNDLQLDDQVALLRAHAGEHLLLGLSRRSMHLKDVLLLGNNCVITKHCPDARLSPNLDISRIGARIIDELVFALRDVNIDDTELACIKALVFFDPNAKGLNEPQRIKTLRHQILNNLEDYVSDRQYESRGRFGEILLILPVLQSITWQMIEQIQFAKIFGVAHIDSLLQEMLLGGMSSSLFHAPHYFHTLRNYLIYSTFEGELADNSPLSPPSLNNYSPTRTMDSSHVTSTLDVLTSPTVNDHLSACMDSNSLDAQMMSPLLENLSSPPTQYSQMRSYPSTRPQEQLSQQGINSNTGNPLHSRSMHSPQLNDTESRNMDMGNGAGNSCMEESMYNSNTIMRPHSTSSAHNLPHMPTPNMQHQHVSASPSQSMQAGALMQHSPPLHRNHPYQRPEQMNTSPNNNGVVPQQLRNPADMTLNEYNSGSAEEMLRRAPLKIRGPDALSSGAGGGGGYMMGGSGLHDAENAYRMTLKQEPETGY

>CcUSP—XP_004536725

MDAGDQEQNFRMGPLSPQEIKPDISILNENNTRAFSPKSASPVSFGSIGLQSMNMANAAAVGVGSNQLQQGAQQQQQQQYPPNHPLSGSKHLCSICGDRASGKHYGVYSCEGCKGFFKRTVRKDLTYACREDRNCIIDKRQRNRCQYCRYQKCLACGMKREAVQEERQRGTRSQNVRSSDDFNPSSSVRDLNIERIIEAEQKSEQLSGDNAIPFLRVGPNSMVQPDYKGAVSHLCQMVNKQLYQMVEYARKMPHFAELQRDDQVLLLKAGWNELLIANVAWCSIDSLDADFATSGGHDTSFGRRSPIRLPQQLFLNHNFSYHRNSAIKAGVATIFDRILSELSVKMKRLNIDRSELSCLKAIILFNPDIRGLKGRNDVEVCREQVYACLDEHCRTEHPGDDSRFAQLLLRLPALRSISLKCLDHLFFFRIMGDKPLDQLFYEQLDTQMC

>CcHR78—XP_004534073

MGASGFKNEDNKAAIATIVHQVGSGNGTSGSSLANTTTATNMSIETCLVCGDRASGRHYGAISCEGCKGFFKRSIRKQLGYQCRGSMNCEVTKHHRNRCQFCRLQKCLASGMRSDSVQHERKPIVDKKEFAGSSSSGSSTSTVTNAAATATALSANNTATGSANLLFGHNLSASVGCNDKSEAPSTSKSSSYIPYNLSLPEIRQAIVNNTDIPVPPLPFQLTFADMRQIIESAKNFQQDKQQAQPLYFSPDLSKPIDEEDDDDESMDNSSTTCLQNLTTNANNNNNTQNLNLNIDPVHSPVTAVGQIHTALDRRVIEKALQLLIPIQNQLERLSSATGNGNMEGSPTIKNEMDDDDMGEDSGAEDEYEIAEAILGVNNAKEFVINECVFENEVLSSAQAAFNLQTPNLISAYLNLHYICETGSRVVFLSIYWLRKIAVFDQLDARTQIKLLRASWPGLLVIALAQVKSLSITTIINTLVANVRQLADVDKIEPQKIRKLSEHIARIHSYIQETLTLELDDMEFAFLRLAILFNPHMLLRKRERNVRDFVRRVQLYVLSSLRKYIASQLSDNEQQAEERFSTLIITLIPLAALESDIIEELFFSNLIGQVQIENMIPYILTLSANNSL

>CcTLL—XP_004521251

MEKDPQKLQALIDIAKVNMDKRYNNPSSPPSRILYNIACEVCRDHSSGKHYGVFACDGCAGFFKRSVRRARQYVCRAKNGGSCIVNKTKRTQCRACRLRKCLDAGMNKDSVQHERGTRSATMRKYAEIYKDVFGSQEMPPEYLYETGQMPFHPMFKGPILTPLPPPPGATAPEVAAPGATATSAELEVAGAVVPPTLASLPVPVPAPVPLAHMPPHLVPQHLQHFQQAQLAALHLQNPHVQQFHRSHPYAQHAEAQAQVHALAQAQVQAQPQAMAMATAQTQANGQAPTQHQQQPHHLLPNPFDTQAVLNLTMAASVAQHGFHPFNFFAPNPGFPGVRALPPTPPLMLNENIKEVAAEQLFKNVSWIRSINAFQELSLNDQVQLLENSWKEFFVLSIAQYLLPINFQHLVFVYETGHPGCEMVNVVKADVLHLQDVLNKICHMNMDCNEFDCLRALSLFRNPAAQSEDSENNSSGTGDSSQNSSTSDESRGLDESAKICNLYENVRKTLQIYVSTTYPTQVSRVQVLESLVPLLSDVSTFMLEELFFRKTIGEVNIVRVLSDLFSQNKI

>CcDSF—XP_020716381

MGTGDRLLDIPCKVCGDRSSGKHYGIYSCDGCSGFFKRSIHRNRIYTCKATGDLKGRCPVDKTHRNQCRACRLAKCFQSAMNKDAVQHERGPRKPKLHPQLHHHHHPHHPHHPHHHHHHHNGLPHHHPGHHLPVSLVTNVSASFNYTSHISTHPVQGFQPPVAHPTAFHHPGHALAQHSALQNGAVATLPPHPQLHFPPHLLPHMHHNLIAEATSKLPSISATATATTAATIVGAVNAAAAVHSTATTSKHNYSSPSPTPSIQSISSIGGRSSLGSESPRVNVETETPSATPPLSTSASPVPSLTDTNVTGGPTSPIIIINGNSNNNNSDASDNNNDSSVNNRHSKAGSGGGSSLTASSGTPSRSTPLSSHSPPAQLGGACASSSSSSSAAAAALYAARQNGFLELLLRPDKCQEIIQYQVHNSLLFPALPQQLIGVDSRLLSWEMLQETTARLLFMAVRWVKCLMPFQTLSKNDQELLLQESWKELFLLNLAQWTIPLDLTPILESPLIKERVLQDEATQVEMKTIQEILCRFRQIAPDGSEVGCMKAIALFAPETAGLCDVQPVEMLQDQAQCILSDHVRLEYPRQATRFGRLLLLLPSLRTIRAATIEALFFKETIGNVPIARLLRDMYVMEPAAQTLVK

>CcSVP—XP_012161984

MITHNSLSVYGGGVMCASPSTAPGFFNPRPQSGAELSAFDLGLPRSMALGVPPPSAWHDPNLGGHLHASAGPGSLPGTAGGSSVGTGGGTTPSSIASQQSAGIKQDLSGLAGTGGQSNTLNQSGHHTIKEDLSSLTAASAAASAAAHHAAAVHHATAAQQNHGQELGVMIKGQQSSSSCLGGGSAGGGGGGGGGGGGGSTTPSSQANSLHSQSSNSDSKQNMDCKQNIECVVCGDKSSGKHYGQFTCEGCKSFFKRSVRRNLTYSCRGSRNCPIDQHHRNQCQYCRLRKCLKMGMRREAVQRGRVPPTQPGLAGMHGQYQLANGDPMGVAGFNGHSYLSSYISLLLRAEPYPTSRYGQCMQPNNIMGIDNICELAARLLFSAVEWAKNIPFFPELQVTDQVALLRLVWSELFVLNASQCSMPLHVAPLLAAAGLHASPMAADRVVAFMDHIRIFQEQVEKLKALHVDSAEYSCLKAIVLFTTDACGLSDVTHIESLQEKSQCALEEYCRTQYPSQPTRFGKLLLRLPSLRTVSSQVIEQLFFVRLVGKTPIETLIRDMLLSGNSFSWPYLPSM

>CcERR—XP_004523856

MSEGSVLHIKQELDVSTSCCSPSTSTQLGVNRSNSYSNGTSASTSSTSSGSVVSGTGTGGNTSTSGNKSSPSVSPERQLCSSTTTLSADLHNVTLAGCEASTPIVLKGNDCGGSNSVSTANSNNGVAGGGSIRDELRRLCLVCGDVASGFHYGVASCEACKAFFKRTIQGNIEYTCPANNECEINKRRRKACQACRFQKCLLMGMLKEGVRLDRVRGGRQKYRRNPISNSYQTMQLLYQSNSTSLEDIKILEILNAYEPDALTVQQQQESHNLNETSGNRSTKVIPSSSVKLELNNGGSGGSHSSNSEILTSSVQPMCIFQNNNCDADEILAVLSDLYDKELVSVIGWAKQLPGFTDLPLNDQMKLLQVSWAEILTLQLSFRSLPFNGRLCFATDVWMDELLAKECGYAEFYYHCVQLAQRLERLSPRREEYYMLKALILSNCDILLDDQSSLRAFRETILNALNDVVCLLRYGSAVSHQQQLLLLLPALRQADGILRRFWRGICHEGKITVKKLFVEMLEPVSR

>CcHR38—XP_004519433

MSLFCNISKYETTITSSISNNIEVRKCISVQKPVQVCAVCGDIAVCLHYGVRTCEGCKGFFKRTVQKNSKYVCLANQSCPVDRRRRNRCQFCRFQKCLIVGMEKEVVRTDLLKGRRGRLPSKQKNKDGFGLYYKISLISALVNGHKETTPDISGLDHTAYSEHQSSYSPLIVSESENVRQFYQILKNSIKSIEDFTYRIPGFRELNAEDRELLFQSASLEMFVLRLSYRTRPNDYKLTFCNGMVMHKKQCEGVFGDWLDHILEFSNRLHTLEIDISSFACICALTVITERHGLQEPKRVEDIQLKIIESLRDYVIFNAKRPRGSYHFSRLLGILPELRSLSIQGLQRIFYLKIEDLVPIPSAIDKMFVASLPF

>CcFTZ-F1—XP_023158418

MDDYSSVPMLSNSGYTNSANNNINNNINNNNNTTVQTQLQTHHHKDQMLSAGNVPATPFIYELPVSHKTNSGNNNNNSHLVKNSTSDYTVFTNCIQQSTLLDQRTVDVSNIDGVSLSSLCDLKFPNSPMADSNFKNISLINIEANNVLVPAKTITTNELITDGNDGVVESTFANILYVKDDIVIEAKDKQSCGVGSGRGGGGSDTAQVTHVTHYLPLKVNNPPAVGEAKFSKATVTVTAVPANAAVAATSAVSALPNNSMMTGMSYNTVETVGSLTNAPATAAAAAAGGSRIGRGGGRGSKSNRGSRVARGHGNGRGGRAAANRQQQQQVKQTEALGSPILASAGGNGEFFKCVSPGNNFNAVILANNEHHNNGSSSRNNNNTTNNTVNNSADNQITTTTILTTTTSPTVMTDTASSKIITHPISNFISSSNTNNNNSISSNHSQNSSASSVRSSCSNNINIISNNNSQINNNNSSSNMPTINAICNNNNNHNVQNTNSIATMSQTRPTVLQCVDEAAAAAAVAAAATALSPTQCPAGAVIMSNCVPEANCNSSSSSNNNNNNNNNTSTLSPSSMEALIESDNQQISQHQQQQQPQQQQPQQLHSQQHQQLLNSSVSIDDDSLSSMDDLLDDQEISEASFGYQDNTSSHSQQSVGGGASGGGGGGSSTSSNATNVVLLNGGNPNASGGYMTLLPPATSSGGHMGISSGGAGPSQGGSGEGIDFKHLFEELCPVCGDKVSGYHYGLLTCESCKGFFKRTVQNKKVYTCVAERSCHIDKTQRKRCPYCRFQKCLEVGMKLEAVRADRMRGGRNKFGPMYKRDRARKLQVMRQRQLALQALRTSIGSVEMKNSPLSPGYQQAYPNMNIKQEIQIPQVSSLTQSPDSSPSPIAIALGQVNTSGGVITTPMNGGNGSGGGGGSGGGGTGGGGGGGGSGNGVNAGANNNNNSNNTSDGLNRSSSGNCHDTGTGSLQNASESKLCFDSGTHPSSTADSIIEPLRVSPMIRDFVQSIDDREWQTQLFALLQKQSYNQVEVDLFELMCKVLDQNLFSQVDWARNTVFFKDLKVDDQMKLLQHSWSDMLVLDHLHQRIHNGLPDETHLNNGQVFNLLWLGLLGVPQLSDYFNELQNRLQDLKFDMGDYVCMKFLILLNPEVRGIVNKKTVLEGQENVQAALLDYTLTCYPSVTDKFRRLLSILPDIHAMASRGEDHLYSKHCAGSAPTQTLLMEMLHAKRKV

>CcHR39—XP_012159996

MSTTKTENATAAGANSTVTAFGTTLASSAFQVPVNMHSHPQVITNGGNNSHSTNGATTTTTTTTTTRNNISVTNIKCEMDESIAASLMPNGNIVPVIAATGSAASYYNGASSGSGSGSGGGAGAGAGGLGVGIRIPVTKAEDSDSETELTNIENLKVTRRVHNSAESNSTADNGNNTVVGGSCYNGSHNKNGPRPMSWEGELSETETTETELMETENSLSNSGMTNMCNMSASGIVDGSMGMTVIKSEATTTTPGHPLAPTPTHPIKPEQMEQLSVEVHGGGSVAAMGPTPVRSGLGSSSSAGSLYSATHTKLAPTQSDPINLKYEPDTSVTGSFSAGANVNSPLLTRNKSATLPLPANPSPDSAIHSVYTHSSPSQSPLTSRHAPYTPSLSRNNSDASHSSCYSYSSEFSPTHSPIQGRHAPPAPALYGNFNGTSLHHSVIYRPITVADNAAAGTAANGSAGVNCSAEAQNLSMDTKVSPLNIGLEGLPASPAGISRQQLINSPCPICGDKISGFHYGIFSCESCKGFFKRTVQNRKNYVCVRGGPCPVSISTRKKCPACRFEKCLQKGMKLEAIREDRTRGGRSTYQCSYTLPNSMLSPLLSPEQQGVAAAVAAAAVAAASQQQQHGRLGAQMSHGMSAQQQQHIMNGLTGGVGGGGAAQTTIKSEQCEESGTSRRATNIPLLLQEIMDVEHLWQYNEAELARLNQPISTSSSSSSNASSTSSSTAANGAGTANPQMTNPLLASAGFGNGENTNPDLIAHLCNVADHRLYKIVKWCKSLPLFKHISIDDQICLLINSWCELLLFSCCYRSIDTPGEIKMSQGKKISLAQAKSSGLQACIERMLNLTENLRRLRVDRYEYVAMKVIVLLQSDTTELHEPVKVRECQEKALQALQAYTLAHYPDTPSKFGELLLRIPDLQKTCQLGKEMLTIKSRDGGDFNLLMELLRGEH

>CcHR4—XP_020717311

MTLSRGPYSELDKMSLFQDLKLKRRKIDSRCSSDGESVADTSTSSPDLLAPMSPKMCDTGGAGAGGGGTHGSGGALSSLPLPTAVVTLTATSTIAPNSVASATVCSSSMTTATSSITGHNVCAPTPPPSSAATAVVVAAGKAATMKGEQQKHQQQQQQQTEIHSSSGAVSAAAASSVMSPPPPSTATVRMSPAPATDLSLPAGRRTPSNTTPTRATPTPPHNNGNASSSCGTSNGGGSSRASPDSMDERPSTTTSTTTTAGTQMSTNGIHGGGANSGLGHAANSASGECMSAAGGGECGVGGNLSVIRSVKEERIAASPTKMLSFQQQQQMQYQQHTQSAPSPTQSKGVTQSQQHVSVLVTPSRIKSELSPQQQQQQQQHVPQMPPSSLPAHLTHHPAYSTSSSAPAASSSTSISGTVTTTSSVSSASLHASSPSPTPLPSAAATSLHSHIHPHPPPPIVQTHHLHQQLTQPQLRKSSPPSGLALPQQNLFNQSMQHLTQQQQLQLLQQAAAMSRPTQMSPNAISSSPSSRHQSSASSPHQHLLQQHHHQQQQQQQQQQQPQHLRIKKEPAQLLAAAAGSLLGNSGGAQRHLHSPHHHHPSSPQHQQQQQQTPPQLPPQALSNIAQLIHPASLQLRHPNRDAAILFRVKNEVHQQVAAAQLMQPGAAAAAAAAAQRMVWVSNARINGVKPEVIGGPLGSLRPGGPAQSPSPHHSSSSSSSQLSPQTPSQTPPRGTPTVIMGESCGVRTMVWSYEPAPPTAGPSPTHSGSNSNSGGGLHQTPPSTPQHQHAQQQQHPHQHQPQPSSMSSLSQQSHHSSQSSLQSASSPSAGSITPTHTPGPSPQNNEEAAQLLLSLGQTRIQDVRPRPHQFRTPHALNMERLWAGDYSQLPPGQIHALNLSAQQQWGNSNATGLNRGLDMPHEPTDEDDQPLVCMICEDKATGLHYGIITCEGCKGFFKRTVQNRRVYTCVADGTCEITKAQRNRCQYCRFRKCIEQGMVLQAVREDRMPGGRNSGAVYNLYKVKYKKHKKSKQQQQQQQQSQQQHMHSPHHQSPLSPHHQQHLLSPQQQLHQHQQQLAAAAAVAAAAQHQHSKLLSATLGQGEMKPMFLGPSIKQEHLQQQQQQQQQQQHAQQQAANQLHSPHHQQLNSPHHTLHGSPHGSLPTHSSSVSSLHSPLSHAGGPTSLPPHTSSSSSSSSSASSGGSSQQGSSHHALHSPLHHLSHGHHLQTNATHPPAHAPQLQLHQQHQQQQQQQQHAQAAAAAAAAASQLQETAIKLPSHLVNGTILKTALTNPSEIVHLRHRLDSAVSSSKDRQISYEHALSMIQTLIDCDAMEDIATLPHFSEFLEDKSEISEKLCNIGDSIVHKLVSWTKKLPFYLEIPVEIHTKLLTDKWHEILILTTAAYQALHGKRNTPSTTTPGGVGGASSTTSTSGRTSIGGAQPPHSPVGSANPPLPKEDPEFVDEVNAHLLTLQTCLTTLMGQPIAMEQLKLDVGHMVDKMTQITIMFRRIKLKMEEYVCLKVYILLNKAEVELESIQERYVQVLRTYLQHSSSQNPQARLTELLSHIPEIQAAASLLLESKMFYVPFVLNSASIRQMLERGLLPIDDDPPKRLMTIIQQQQQQEQQQQQQQQQQEQPREDDDEDEEVNKVEHHQQQNASNLMKARRRVATQMAERQQRQKEELKKQQQEQLQQRQTSQQQRLEEQQLPQPARFCLPMLELPRIKVENVQKELLYSMEEDTADSEEEEEELRIMEERRQLERRLKVEESSAQLPAMSRLMEPTTSILKTSLLAGAAATLATLTAAAEQVNRTSLSPQAHGDGGGGGGGGGHSRGLGARTLSAGRRTPAVAENSLIESKRLLTQQQQQQQQQQQQDQCETPAAPIMPYKSILQTALMGERPLRSETPPPPPSPQSTVMRAAGALASLQAQTTPPPPPPPHVLKTAVTLASLSEIAAARQQQEEEEERLMREQQQQQQQQREHLLLQQTLPLKKKKSFLQQKLTATCNMLTTAATILQQQQQQQQQQQQHKQLPQNTITILPVEANSNKNNNLNTAMSATNAATTAITMPTTVTKFVLNNVIASGAGGSGGGAGVLRCTASSATTAGAAITTTKRSTATFVNNTILRPTTLTLSSSGSGSQSRMSGVQSATEASNKSNASNNIVSNIKNIANSCQAIATQTIKIPSVRTATVTTQTQTDEMPTSAAPATATATSGEQQQAPKQTPLHALSASSHVITRKLSTVLQQQQQQYATTNSGNSTSATLLTLPVQQQSISRRRLSPQRQTTLVTATTNASTICAAAAPPTTAISTATAASPPSTAVTVVVFKTLLPDD

***Tribolium castaneum (Tc)***

>TcKNI—NP_001121967.1

MNQTCKVCGEPAAGFHFGAFTCEGCKSFFGRSYNNLSSISECKNNGECVINKKNRTACKACRLRKCLMVGMSKSGSRYGRRSNWFKIHCLLQEQQQQQQAGGGGIQTGLKPPQKTPSPHQHLGLGLLNQPFGHPLMHHITKTKEELMLLGLDEYKNSASPSVSSPESHNSDSSVEISDARRIPLFPGLLPPTFLPPHGLLFPPGYPPLYPGLLQPTNNNNRLMRNHNPGVEAFNKRVFLDAVLQSQRSPTPEVEAPPPVASPLQEDPIDLSMKTMSERGSSPAHSDRSDSVVTEQGAGSEADEESDCESEKELKRIKLVRPAPLDLTTKV

>TcEGON—NP_001107839.1

NPEAGLETRILIMCASTANEMMNQLCKVCGEPAAGFHFGAFTCEGCKSFFGRTYNNISSISECKNNGECVINKKNRTACKACRLRKCLLVGMSKSGSRYGRRSNWFKIHCLLQEQQQAGANMAAAANLLRPHPYLSPLFHSAAPRTVSRETKSSESDSGASSADPDDELRSSSAFSYIKPSSERDYFSVKKLPSPASDSECFDRRKFISALVNAPSVSPASLPSISPSGGFLPKWLPPLQGFSDPSAWRDLWARGPVTTAAAAPDQVEPIDLSLKSAPKGTALPAPDSDQELCIDVGVDEPLKPVPLDLTLDRQTTDVSN

>TcE75—XP_971362.2

MILSPDSSGQLPESTTDSNTMASHREPALNLEFDGTTVLCRVCGDKASGFHYGVHSCEGCKGFFRRSIQQKIQYRPCTKNQQCSILRINRNRCQYCRLKKCIAVGMSRDAVRFGRVPKREKARILAAMQQSSNSRSLEKAVAAELEDEQRLLATVVRAHIDTCDFTREKVEPMLARAREQPSYTACPPTLACPLNPNPQPLTGQQELLQDFSKRFSPAIRGVVEFAKRIPGFSLLAQDDQVTLLKAGVFEVLLVRLACMFDTQTSSMICLNGQVLKRDSIHNSSNARFLMDSMFDFAERMNALRLSDAEIGLFCSVVVIAPDRPGLRNTELIEKMQNKLKAALQMVVNQNHHGQTHIIDELMKKVPDLRTLNTLHSEKLLAFKMTEQQQMMQQQHQQLWNTPMEEESSSKSPTASSWSSSSDVTMDEAKSPLGSVSSTESMCSSEITSLNDYHQLGQHHVSSNVTNAPLLAATLSGGLCPHRHRANSGSTSSGDDELAATSHLIHNGLTITPVARPHTHPRFRKLDSPSDSGIESGTEKVDKHSAPTSLCSSPRSSMEENHIVEEMPVLKRVLQAPPLYDTNLLMDEAYKPHKKFRALRNKDSAEAEPAVVMSPQPQVQHQQQQSSLSSTHSTLAKSLMEGPRMTAEQMKRTDIIHNYIMRDEHPVGAASPMVTAAPTSCPYKVNNGLLQPAWATSVITTTGRHQQQCSSPQEYNRMYLQSPHSTSPAPPSSPLKIVELQVDIADSQQPLNLSKKSPSPSPRPMKVVTLEV

>TcE78—XP_015834061.1

MEPSSSTPSSPGNADSTFTSVKQEPTSNPASPLEASSPTSIALRNKEQHYVAADSNSSSTTKSFVPCKVCGDKASGYHYGVTSCEGCKDGKCLVIRLNRNRCQYCRFKKCLSVGMSRDSVRYGRVPKRSRERSTEESSRVTTTDADQSDSETKQLAVYDVILTVSQAHHANCGYTEEHTRNLVRKPVVVPPSSPADSEVASSTAEALEQERCWLWQQFAANITPSVQRVVEFAKRVPGFCDLGQDDQLILIKIGFFEIWLSHVARLTSNTVMMFDDGTTVTRQQLEIMYDADFISSVMHFANTFNSLALNDTEVGLFSAVVLLTADRSGITDVKSIEHHQDKLIEALKVQVGRNHANEPQLFSSLLIKLPELRNLGAKHSAHLDWFRMNWTKLTLPPLFAEIFDIPKSEDDLQ

>TcHR3—XP_008195824.1

MFEMWNNVSSRLEQVQQSQTQTQQPHTSSGSIKAQIEIIPCKVCGDKSSGVHYGVITCEGCKGFFRRSQSSVVNYQCPRNKNCVVDRVNRNRCQYCRLQKCLRLGMSRDAVKFGRMSKKQREKVEDEVRFHRAQLRAQSDSAPDSSVFEQQTPSSSDQLHHSYNGGYTYNSDVSAYTPAYYTNTTQVQANSMGYDISADYVDSTTAYDPRPGLDTLNDTGSLMANVVTTDPSQISELLSKTISDAHTRTCLYSLEHIHEMFRKPHDLSRLLYYKNMAHEELWLECAQKLTTIIQQIIEFAKMVPGFMKLSQDDQIVLLKAGSFELAIIRMSRYLDLSQNCVLYGDTMLPQDAFFTSDTAEMKLVTCVFEVSKSIAELKLTETELALYSACVLLSPDRPGLKGIGEISRLSQAVVRALRLELDRNHSMPIKGDVTVCDALLTKIPTLREISMLHMDALGKLKRSAPHLEFPALHKELFSVDS

>TcECR—NP_001135390.1

MKRRWSARVAPEESSSEVTSSSALVMSPANSLASTDIGDVDLEFWDLDLNAQNHPRGLTIIQNGYGLPGHTIIASNHHLAKSDTSSASGREDLSPSSLNGYSADSCDSKKKKGPTPRQQEELCLVCGDRASGYHYNALTCEGCKGFFRRSITKNAVYQCKYGNNCEIDMYMRRKCQECRLKKCLSVGMRPECVVPEVQCAVKRKEKKAQKEKDKPNSTTNGSPDVIKVEPELSDSEKTLCNGSKGISPEQEELIHRLVYFQNEYEHPSEEDVKRIINQPMDGEDQCDVRFRHITEITILTVQLIVEFAKRLPGFDKLLREDQIALLKACSSEVMMFRMARRYDVQTDSILFVNNQPYSRDSYNLAGMGETIEDLLHFCRTMYSMKVDNAEYALLTAIVIFSERPALIEGWKVEKIQEIYLEALRAYVDNRRKPKPGTIFAKLLSVLTELRTLGNQNSEMCFSLKLKNKKLPPFLAEIWDVDLKT

>TcHR96—XP_968487.1

MKSKVDTKVCGVCGDKALGYNFNAVTCESCKAFFRRNALVQKQFKCPFNDQCEITTITRRFCQKCRLDKCFAIGMCKDLIMSEEDKHLKRQKIEENRARKRSKIHGFKSGFKKVKKESVEADSQDSQSGYSFIDSDSSKDMGYQPNGTPNPVTVDLGNERLVTSSDDVSMIRNMLNNTPNSATGSKSVDDSALISLINNNDVNCNNNDPTSSSKSTLDVTKDVVEDVHRVSIEPNSIESILCEAIKLEFEAYSSIAQCHSNSRELNDAERAKLNELIVANKALLNPLDDELNTLIGNDYKFRGNPQQALETDPALVDVINLTAIVIRRLIKMAKKINAFKNMCQEDQVALLKGGCTEIMILRSAMNYDPTKQTWKIPHSQETFNLNVDILKLAKGNVYQEHERFINTFDPKWRSDENIVLILCAITLFTPDRPRVVHQDVIKLEQNAYYYLLRRYLESIYPGCEAKSTFLKLIQKIAELHRFNEELINVYLDVNPSQVEPLLIEIFDLKH

>TcHNF4—XP_008196276.1

MPSSYCSTSPGEISISDGEVDGTDYLLSSTIKLDENFFQILDTEMTMAASNNSLTATLSSGSAAGVAQMSQHCAICGDRATGKHYGAASCDGCKGFFRRSVRKNHLYTCRFSRNCIVDKDKRNQCRYCRLRKCFKAGMKKEAVQNERDRISCRRPSYEENNQNNGLSVGSLLNAEMLSRQVGAALEQMGPTPVNDYDLSNKQLASINDVCDSMKQQLLILVEWAKYIPAFTDLQLDDQVALLRAHAGEHLLLGLARRSMHLKDILLLGNNCIITKHSPDSRVSPDLDISRVGSRIMDELVKPMTEVQVDDTEFACLKAIVFFDPNAKGLSEPARIKALRYQIQINLEDYISDRQYDSRGRFGELLLTLPALQSITWQMIEQIQFAKLFGVAHIDSLLQEMLLGGK

>TcUSP—XP_008192984.1

MMKKEKPMMSVTAIIQGAQNWSRSLPNFGPPHMSLALSLDQNLSMGSLGAPQSPLDMKPDASTLGQFSPQGPNSPVSFSMGGHGTLLSPSGNTPNKSSTSPYPPNHPLSGSKHLCSICGDRASGKHYGVYSCEGCKGFFKRTVRKDLSYACREEKNCIIDKRQRNRCQYCRYQKCLNMGMKREAVQEERQRTKDRDTSEVESTSNLQADMPLERIIEAEKRVECNDPLVALVVNENNTTVNNICQATHKQLFQLVQWAKLVPHFTSLPLTDQVQLLRAGWNELLIAAFSHRSMQAQDAIVLATGLTVNKSTAHAVGVGNIYDRVLSELVNKMKEMKMDKTELGCLRAIILYNPDVRGIKSVQEVEMLREKIYGVLEEYTRTTHPNEPGRFAKLLLRLPALRSIGLKCLEHLFFFKLIGDVPIDTFLMEMLEGTTDS

>TcHR78—XP_001813130.1

MDIEHSIKSEILSDKIRNNNSNNLCLTVELCVVCGDRASGRHYGAISCEGCKGFFKRSIRKQLGYQCRGSKNCEVTKHHRNRCQYCRLQKCLACGMRSDSVQHERKPIIDKKDYSNNIGNSYNSNSVNKIFIRKDLSTDSPGLLPAPFNPCDLGLQFLNKRIGSSGSSELPYHLSPSQASIEDDVSMDSTNTGAGELSDALIMARDKQLISKALDTMARVQCLNGSDLSSLASSEEKCYDYDGPILSEQHISFNLQIPGPVPPYLNIHYICESGSRLLFLSIHWTRNIPAFQYLSTETQITLLRGCWAELFTLGLAQCSQTLSLSTILSALISHLHTLIAQDKMSATKVKQVSDHIVKLKDYATTMNLLNVDEHEYAYLKAITLFSADQPDVLLKKHVEKLQEKSFQGLKNYVHSSFPDDVDRFPRLLLRLPPLRGLEPLVLEELFFAGLIGQVQIDSVIPYILRMGNGMPTPGNRHVKSEQIDDFMCK

>TcTLL—NP_001034502.1

MSEMQSVEGAMVHHLEPHRMQIKPQSPSSSSRILDIPCKVCGDFSSGKHYNIFACDGCAGFFKRSIRRNRQYVCKAKDEGSCIIDKTHRNQCRACRLKKCQNVGMNKDAVQHERGPRNSTLRRQQMSSYYNESRVMMSPPGNVLNLTMPKYEPNPSIIDPGPALPPTGFLCNNYPPLPQVPPLPLPPIFPPTMINPSAICESAAQLIFMNVQWVRSIPAFTCLPLSDQLLLLEESWLDLFVLGAAQFLPLMDFSVLVEACGVLQQEPHRRDAFLKEVADFQETLKKISQFQLDAHEFACLRAIVLFKTSFEKPSSSSNQEKTTTESAKISVIQDDAQMRLNKHVTTTYPKQPLRFGKILLLVSSTFRTISGRTIEDLFFKKVIRDTPIVAIISNMYKNQILGNNNV

>TcHR51—XP_015836760.1

MDCSVSQAGSILPTLHQPCSPEHHSHHPNDLRTSPGSVDCYSSEGDHPLALCLPNSQTLVQASRTPPPPWSIPTKKGTPGLICVVCGDTSSGKHYGILACNGCSGFFKRSVRRKLIYRCQAGTGRCIVDKAHRNQCQACRLKKCLAMGMNKDAVQNERQPRNTATIRPETLRDMSAEQERALREAAVAVGVFGPPVPLTMALTSARYSHNLLPSVPPPLPNREPTPTKDTSDNDEESIDVTNEEEDTPSSGCGVSSSSGPLRPEPQPALTSGLYPSTHETIYETSARLLFMAVKWAKNLPSFASLPFRDQVILLEEAWSELFLLNAIQWCMPLDVSASPLFNVNEHVKNGHSATDVRILADTLMRFKAIHVDPAEFACLKAIVLFRSETRGLKDPSQIENLQDQAQVMLWQHCRTQLPGQVARFGRLLLMLPLLRIVPASRVEAVFFQKTIGNTPMEKVLCDMYKN

>TcDSF—XP_008201026.1

MGTGDRLLDIPCKVCGDRSSGKHYGIYSCDGCSGFFKRSIHRNRVYTCKATGDMKGRCPVDKTHRNQCRACRLSKCFQASMNKDAVQHERGPRKPKLQASSNQQMQPSPVGHSLQQHMTTVGDKLPTSSSSLHFAPSIFGPPRPLKALTLPPPPVPSPTHIDALPPSMFHPPTLPPPPGLLHILMSAEKCQELVWGTKLSQMESPSSSETNSGINISPGIPVAQPPPIPPLPLTPTWEMLQETTARLLFMAVRWVRCLAPFQTLSKHDQLLLLQESWKELFLLHLAQWSVPWDLSALLGCPQARERLPADIHTATEIKTIQEIMCRFRQISPDGSECGCMKAIILFTPETAGLCDVQPVEMLQDQAQCILGDYVRTRYPRQPTRFGRLLLLVPSLRAIRSLTVELLFFKETIGEIPITQLLGDMYYMEKCTSGPP

>TcHR83—XP_008200485.1

MGRTLPTPVPCRVCGDRSFGKHYGVYCCDGCSCFFKRSVRRNIIYTCISGEGRCIVDKARRNWCPYCRLQRCFSVQMNVAAVQEERGPRKPKLVSRKPSYSINSESFVYSTVSRPLPPETNPIQELAAQILLVAIKQARCNSGFGLLNRASQNLILSHLWAPLFLLRAAHWPSESADLFPGVKGTFRVVRQLKLNLTDLEIVENILLCRADILDDVEQITLALNVLTRALDELAVRTVLERRRFADILLALPVLFVPSAVVLHSLLFKPVIGAVPIETVISTI

>TcPNR-like—XP_001814012.1

MASINKSFLYGICFASITWIISLYLYINLTNNEPTTTHIPFKFAKTGHPLNSFDRDYKVSKSYRLRRKFFNQGTNSKQLINKLQPVYPKLSTDKNELSQLGLVKNIDDQRKKDEGYKKHAYNVLISERLSYHRDVPDTRNELCKNISYSADLPTAAIIICFYNEHYYTLLRTVHSIIDRTPASVLKEILLVDDFSDLENLHENLSTYITKNFDDRVKLIKTERREGLIRARLFGARRTKQDVIIFLDSHIEVNVGWIEPLLQRIKDNYTNVAMPVIDIINADTFAYTASPLVRGGFNWGLHFKWENLPKGTLSTKMDFIKPIKSPTMAGGLFAMSRKYFTDLGEYDAGMNIWGGENLEISFRIWMCGGRLELIPCSRVGHVFRQRRPYGAPDGQDTMLHNSLRVANVWMDSYKEYFLNHRPDAKRIDFGDVSSRVQLRKELKCHDFDWYLKNVYPELALPTDNEERLKKKWTALESDKFQPWHLRKRNYVDQYLMRLSNTSLCVQAAKDIKSKGSNLVLRKCVRTKNQMWYQSDKNELVLGQLLCLQAGKKLPILYKCHEMGGDQEWNHKGENKSAIYNIAAGTCLGAEEQKLGAFITMKLCSDPNLISWDIVR

>TcSVP—XP_967537.1

MALVASSWRESSLVNPIPTSLNSLPNGLNPLPPTANLTALPANINPITLENPDLALVNRQTPSQNSQNSATSADKNQNIECVVCGDKSSGKHYGQFTCEGCKSFFKRSVRRNLTYSCRGSRNCPIDQHHRNQCQYCRLKKCMKMGMRREAVQRGRVPPSQPALPGLPNQFLNSTDSVTSSLSNHTYLSSYISLLLRAEPYPTSRYGQCIQANNIMGIDNICELAARLLFSAVEWARNIPFFPDLQVTDQVALLRLVWSELFVLNASQCSMPLHVAPLIAAAGLHASPMCADRVVQFMDHIRIFQEQVEKLKALHVDSAEYSCLKAIVLFTTDACGLSDVAHIESIQEKSQCALEEYCRSQYPNQPTRFGKLLLRLPSLRTVSSAVIEQLFFVRLVGKTPIETLIRDMLLSGSSFSWPYM

>TcERR—XP_015836766.1

MMTDPGVGGDVSPGVPQIKKELDGFQECSPSNSDMYSPTTTVMHDTGIDFQETNHYDSSIRSPGSPERQYCSSTTQPHTDSGMGHMEDEMKEEDSPRRLCLVCGDVASGFHYGVASCEACKAFFKRTIQGNIEYTCPASGECEINKRRRKACQACRFRKCLRSGMLKEGVRLDRVRGGRQKYRRNPDSPYQVQVVSAQRPMLQLEDIKMLDALAICEPDCLEINKALENSKHRTLSILSDLYDRELVGIIGWAKQIPGFTDLSLNDQMRLLQSTWAEILTLTLAYRSVPLIQLARLKFANDFTLDEKQSRDCGAIELYQTCCHVVERLDNLSILKEEYYLLKALVLANSDVRLDESHSLKKFRDSILSALSDTVGILRPTGVLHQIQQLLLCMPALRQADQVVRRFWSDVHQQELVPMNKLFLEMLEAYCR

>TcHR38—XP_008194320.1

MRSGPPTLRQHFRLQQHFNAPQQTHTDSNNNSPAYNFHLQNKCTVLVQGEVSSAPLVAASAQPSAPTSPQSSMLLLQTQTPFGSSSFADLLSAPFSDDPSSVLPPEDLDPFPDVALQSQPASPAPLPSFQETYSVRYNAPFKMDEECFNVSYHHPPHHVSAPHHYDYQQLQYTASSPYYPPPQTCSPGFDTLISQETYSLPAYQTATSELHVSTALSPRQRRASLPLQRSESTSSSESPKLRMPGPSASSSAASSPGGIAEPPSKGPTPPSPSQLCAVCGDTAACQHYGVRTCEGCKGFFKRTVQKGSKYVCLADKACPVDKRRRNRCQFCRFQKCLAVGMVKEVVRTDSLKGRRGRLPSKPKSPQESPPSPPVSLITALVRAHVDTTPDLANLDYSQYLEPSPIEPVISEAEKIQQFYTLLTTSVDVIKNFADKIPGFQELCPEDRELLFQSASLELFVLRLAYRTHPNDTKLTFCNGVVLDKQQCQRSFGDWLHAIMEFCSSLMAMEIDISAFACLCALTLITERHGLKEPHRVEQLQMKIISSLRDHVTYNAEAQRKAHYFSRLLGKLPELRSLSVQGLQRIFYLKLEDLVPAPPLIEKMFVASLPF

>TcFTZ-F1—XP_015833964.1

MHEETASMSVPGPVAVSAGQSEGSPTPTAVVVNAAEVDLEVAPDADNVELQVPFAANSGGASAISGVDMGASYQISGPATSLTAGDLPDTKDGIEELCPVCGDKVSGYHYGLLTCESCKGFFKRTVQNKKVYTCVAERSCHIDKTQRKRCPYCRFQKCLEVGMKLEAVRADRMRGGRNKFGPMYKRDRARKLQMMRQRQLAAQTLRGSSLGDAMYSSQPGTSPFANIHIKQEIQIPQVSSLTSSPDSSPSPIAVALGQVNTSNLVQQASSQQPALQIVGVPGGPSSMVMGPDNKLWGSANSTTTSPHSLSPKAFQFDTVVQGGSAPPSSKVSPLIRDFVQAIDDREWQNSLYTLLQNQTYNQCEVDLFELMCKVLDQNLFSQVDWARNSVFFKDLKVDDQMKLLQHSWSDMLVLDHMHQRMHNNLPDEMTLHNGQKFDLLSLGLLGVPSLADHFTDITAKLQELKFDVSDYICVKFLLLLNPDVRGITNKKHVQEGYEQVQQALLEYTVTCYPQIQDKFNKMMQLLPEIHSLATRGEEHLYHKHCSGSAPTQTLLMEMLHAKRK

>TcHR39—XP_008191947.1

MAADMRSPNVSVTPINMEPPYPSTPITNCDLPTGEPQRTRSAPHPGLLTNGDVLDSSDAEVAQIDFQCAVNMRHKKKRDGADVPRPMSWEGELSDHERDMCVDDDNGSQVPENLSLSQPKLNTIVNLDSKESPIMRLKQPPMGTYSLHSTPSNSPLLSQRHPVPIIGGPKDLIHKDIMHKALNVPPTPSPDSAIHSAYSVFSSPNQSPLTQRHITLTPNLSRNNSDASHSSCCSYSSVSVSVSPTQQFSPTHSPIQGRHMHSIHNSPLHHNVLYRSLQEDPSVQYTDHDGLDEAEDKTGLVHTSLPAQPGISRQQLINSPCPICGDKISGFHYGIFSCESCKGFFKRTVQNRKNYVCLRGAACPVTVATRKKCPACRFDKCLQCGMKLEAIREDRTRGGRSTYQCSYSLPPSLTNHAELRASSTDLRQNMGETHHGLTVKLEPSDSSVSHHKRGTLPGSRGVPALLQEIMDVEHLWHYNEVELNKLSTEGHLGKEGGSGAVHNLSNHPLLAGTALAGSTDTNPDFVANLCNIADRRLYKIVKWCKSLPLFKNISIDDQICLLINSWCELLLFSCCFRSMSTPGEIRVSLGKSITLNQARDMGMQACIERMLNFTEQLRRLRVDQYEYVAMKVIVLLTSDTSELREAEKVRASQEKALKALQDYTLSHYPDSPAKFGELLLRIPELQRTCQVGKEMLTIKSKDGDGPSFNLLMELLRGDH

>TcHR4—XP_974320.3

MSEAVVSPPSPDSTPHHPPEPTLDSVSSRLEDSGVFDGGGVIRPLPRSQSPPLPAPAAGPRQPPRPHSSPHSPGMAPPEPRSVIISPLWKQRINGVKPELIGGSPARPPGAAPARQTPTVIMGEAGGVRTMIWSQPETPPHATTSWGSAPEESAAQMLLNLGQDRIRPVARNVPPQAPSTAHFAAAPLNMERLWAGDLRQLPLSQTPLNLSSPPPVYTSGAEKNVGESTSESQEAAEEEEQPMICMICEDKATGLHYGIITCEGCKGFFKRTVQNRRVYTCVADGNCEITKAQRNRCQYCRFKKCIEQGMVLQAVREDRMPGGRNSGAVYNLYKVKYKKHKKPACKQPPKAAEKNILSQQFKMEQPSSIPANLVNGTILKTALTNPSEVVRLRQRLDSAVSSSRDRNFSIEYSLSMIKTLIDCDEFQDIATLQNLDDLLDHNTDLSEKLCHIGDSIVYKLVQWTKRLPFYLELPVEVHTRLLTHKWHELLVLTTSAYQAIHKAGDQLTTIIKTDFNHEVETNLCTLQSCLTSMMGREITIEQLRQDVGLMIEKITHVTLMFRQIKLTMEEYVCLKVITMLNQAKPASSSGNSELESIHERYMTCLRVYTQHMYPQQTTRFQDLLGRLPEIQSAASLLLESKMFYVPFLLNSAIQR

***Bombyx mori (Bm)***

>BmE75—NP_001037042.1

MQCYPKLSPKREPPEGLYEIEMLPGARRLELPAPPGKEFRAPVLLAGPSLAPTHSVIQCMRPPPPPPPPPPPRLLKPPSFEEPSSSIPDLEFDGTTVLCRVCGDKASGFHYGVHSCEGCKGFFRRSIQQKIQYRPCTKNQQCSILRINRNRCQYCRLKKCIAVGMSRDAVRFGRVPKREKARILAAMQQSSSSRAHEQAAAAELDDAPRLLARVVRAHLDTCEFTRDRVASMRARARDCPTYSQPTLACPLNPAPELQSEKEFSQRFAHVIRGVIDFAGLIPGFQLLTQDDKFTLLKSGLFDALFVRLICMFDAPLNSIICLNGQLMKRDSIQSGANARFLVDSTFKFAERMNSMNLTDAEIGLFCAIVLITPDRPGLRNIELVERMHSRLKACLQTVIAQNRPERPGFLRELMDTLPDLRTLSTLHTEKLVVFRTEHKELLRQQMWNEEEGVSWADSVVEESARSPIGSVSSSESGEVPSDCGTPLLAATLAGRRRLDSRGSVDEEALGVAHLAHNGLTVTPVRPPPRYRKLDSPTDSGIESGNEKHERIIGPGSGCSSPRSSLEEHTEDRRPTAPADDMPVLKRVLQAPPLYGGTSTLMDETYKPHKKFRAMRRDTGEAEARPVQPTPSPQPLHPHPASPAHPAHSPRPPRISLSSTHSVLAKSLMEGPRMTPEQLKRTDMIQQYMRRNEAGSSVEGCTLRTGGLLTCYRGASPAPPPVLALQVDVTDAPLNLSKKSPSPPRSYMPQMLEA

>BmHR3—NP_001037012.1

MLNMFDMWNSVSKLEAQSNVQQSQQPHTSGGSIKAQIEIIPCKVCGDKSSGVHYGVITCEGCKGFFRRSQSTVVNYQCPRNKACVVDRVNRNRCQYCRLQKCLKLGMSRDAVKFGRMSKKQREKVEDEVRYHKAQMRVQADAAPDSVYDAQQQTPSSSDQFHGHYNSYPGYGSPLSSYGYNNAGPALPSNMSGMQPQPPAQPPYEVSGDYVDSTTTYEPKQTGFLDADFISHVEGDISKVLVKSLTEAHANTNPKLDYIHEMFGKPQDVSKLLFYNSMTYEEMWLDCADKLTAMIQNIIEFAKLIPGFMKLTQDDQILLLKSGSFELAIVRLSRLIDVNRDQVLYGDVVLPVRECVHARDPRDVALVQGIFEAAKSIARLKLTETELALYQSLVLLWPERHGVMGNSEIRCLFNMSMSAMRHEIEVNHAPLKGDVTVLDTLLAKIPTFRDLSLMHLGALSRFKATHPHHVFPALYKELFSLDSVLDYTHG

>BmECR—BAA07890.1

MRRRWSDNGGFPLRMLEESSTEVTSSSALGLPPAMVMSPESLASPEYGALELWSYDDGITYNTAQSLLGACNMQQQQLQPQQPHPAPPTLPTMPLPMPPTTPKSENESMSSGREELSPASSINGCSADADARRQKKGPAPRQQEELCLVCGDRASGYHYNALTCEGCKGFFRRSVTKNAVYICKFGHACEMDMYMRRKCQECRLKKCLAVGMRPECVIQEPSKNKDRQRQKKDKGILLPVSTTTVEDHMPPIMQCDPPPPEAARIHEVVPRYLSEKLMEQNRQKNIPPLSANQKSLIARLVWYQEGYEQPSDEDLKRVTQSDEEDEESDLPFRQITEMTILTVQLIVEFAKGLPGFSKISQSDQITLLKASSSEVMMLRVARRYDAASDSVLFANNKAYTRDNYRKAGMAYVIEDLLHFCRCMFAMGMDNVHFALLTAIVIFSDRPGLEQPSLVEEIQRYYLNTLRIYIINQNSASSRCAVIYGRILSVLTELRTLGTQNSNMCISLKLKNRKLPPFLEEIWDVAEVPTTHPTVLPPTNPVVL

>BmHR96—XP_004933007.1

MDNIGENKTENVSQKKELPLNLQKICLVCGDKALGYNFNAISCESCKAFFRRNALASKEFKCPFTNNCVITVVTRRFCQKCRLEKCFAVGMVKEFIMSEEDKAEKRRKIEENRARKRKSDCDEAVSSSKSMKKDDENLTSNYPPESIQYDVLNSTTCSPSSTVQSPLNNDMESSIQSPPMYNYAPIPPVQPEYNMKVFPIDEKQIMDQALYEQRMMDSYKMYDNVESNTYMEPPKQNSIRQILTNGDTATAFKETKQQHVCEEIPSTSTDPDVNKARDILQDVERIEPNSMESILCEAIKLEFESYASVNSCSGSSRELNEVERAKLNELIVANKALNAPIDDDISQLVDSASAEPGGKHDPRLIRLVNLTAVAIRRLIKIAKKISAFKNMCEEDQVALLKGGCIEMMVLRSTMTYDGQRNQWKIPHCQEQFGRIGTDVLKLAKGDIYRSHEAFISTFCARWRTDESVILILSAILLFAPHRPRLVHRDVVKLEQNSYYYLLRRYLESVYPGCEAKSTFLKLIQKVLELRKLAEEITDVYLDVNPLEPLLMEIFDLKHHGGRD

>BmHNF4—NP_001037474.1

MRSDVLAPSELEGTDYLLSSTMRLEDSFLQILDSDMQLETSSSEASAASSTTISQHCAICGDRATGKHYGASSCDGCKGFFRRSVRKNHLYTCRFSRNCVVDKDKRNQCRYCRLRKCFKAGMKKEAVQNERDRINCRRPSYEEPTQANGLSVVSLLNAELLSRKVIDETVNVSDAEINNRKLAKINDVCDSIKQQLLILVEWAKYIPAFTVLHLDDQVALLRAHAGEHLLLGCARRSLHLKDILLLGNNCIITKHNIDGRMDIDISMIGMRVMDEIVKPLREIDIDDTEFACLKAIVFFDPNAKGLSQPQKIKQLRYQIQINLEDYISDRQYDGRGRFGELLLCLPPLQSITWQMIEQIQFAKLFGVAHVDSLLQEMLLGGATTEAVLEEPSPEPAAASPSPPLVPLVPLVPQLPQLPGNDSAFLEPMPFKQEPNI

>BmUSP—NP_001037470.1

MSSVAKKDKRTMSVTALINRAWPMTPSPQQQQQMVPSTQHSNFLHAMATPSTTPNVELDIQWLNIESGFMSPMSPPEMKPDTAMLDGFRDDSTPPPPFKNYPPNHPLSGSKHLCSICGDRASGKHYGVYSCEGCKGFFKRTVRKDLTYACREDKNCIIDKRQRNRCQYCRYQKCLACGMKREAVQEERQRAARRTEDAHPSSSVQELSIERLLELEALVADSAEELQILRVGPESGVPAKYRAPVSSLCQIGNKQIAALIVWARDIPHFGQLEIDDQILLIKGSWNELLLFAIAWRSMEFLNDERENVDSRNTAPPQLICLMPGMTLHRNSALQAGVGQIFDRVLSELSLKMRSLRMDQAECVALKAIILLNPDVKGLKNKQEVDVLREKMFLCLDEYCRRSRGGEEGRFAALLLRLPALRSISLKSFEHLYLFHLVAEGSVSSYIRDALCNHAPPIDTNIM

>BmSVP—BAB55582.1

RRASSRPVTATGIITHGALVYGGVGGPGLCPPRGLDHPPLELGFRGSWREDEHLPSTPASQAASTQSGSSATDKGQNVECVVCGDKSSGKHYGQFTCEGCKSFFKRSVRRNLTYSCRGNRSCPIDQHHRNQCQFCRLRKCLKMGMRREAVQRGRVPPSQPPGLGLPGQFTLGNGDPGAGLNNHPYLSSYISLLLRAEPYPASRYSPCVQPTNVMGIDNICELAARLLFSAVEWARNIPFFPELQVTDQVALLRLVWSELFVLNASQCSMPLHVAPLLAAAGLHASPMAADRVVAFMDHIRIFQEQVEKLKALHVDSAEYSCLKAIVLFTTDACGLSDVTHIESLQEKSQCALEEYCRTQYPNQPTRFGKLLLRLPSLRTVSSQVIEQLFFVRLVGKTPIETLIRDMLLSGNTFSWPYMATM

>BmERR—ANS60466.1

MMSAVSGEPMLRRVKQETDPSPQYSPPEPQPPHPQPLHPRPMPLMQPEVPMPLQQLELETKDVKFCVSPEDGTIVGNTSPEQQHCSSTTAAAASGNRDEDTPRRRCLVCGDVASGFHYGVASCEACKAFFKRTIQGNIDYTCPAANECEINKRRRKACQACRFRKCLRTGMLREGVRLDRVRGGRQKYRRAPEQAQPRVQLDDIKVLEALLSYEPEPFACSAAPPGVTEPAARTLALLADLYDRELVEIIGWAKQIPGFTDLQLNDQMRLLQSTWAEMLSLMVAYRSMSSGANLKLRFASDLTLDEQEARDIGATDLFLQVSGVVRRLERAGAVREECVLLKALVLANSEARLDEHLTLRRFREQILQALNEAVAVLRPYGANSALQQLLLVLPALRHADVAVRRFWAAVHRDRRAPMNKLFVEMLEACLR

>BmHR38—XP_004931089.2

MRGALLTPSSQICGLRQFQYTRPSAIESRSPSAFTPNSSSMLLLQTHSNYGSSFSDLLSPQYQEDSTEILEENLDPFPDVEFHAPIVPEFKSQRDTSFSETSSPIPGPALPSFEETYSVRYPKHEMAEFGIKMDEDCYNVSAYSHQGHTSTQLLYQYHQPPIPYVPSTYYAPPQPCSPTFDTVTPNQDSYSLPPFPSTVDLHISAEQAARQRRSSLPVQRSESNSSNDSPKLHGTRVHCMQPSTPNSASSSPGVAPADNTGPRAAPSSPSQLCAVCGDTAACQHYGVRTCEGCKGFFKRTVQKGSKYVCLAEKSCPVDKRRRNRCQFCRFQKCLAVGMVKEVVRTDSLKGRRGRLPSKPKCPQESPPSPPISLITALVRAHVDTSPDFANLDYSQYREPSPLEPPMSDLEVIHQFYSLLTTSIDMIKLFAEKVPGYGDLCPEDREQLFASARLELFVLRLAYRTRLEDTKLTFCNGLVLDKRQCQRSFGDWLHAVLDFSNTLHSMDIDISTFACLCALTLITDRHGLKEPHRVEQLQMKIIGCLRAHMPGGGGSSSGAPHFSRVLGALPELRSLSVQGLQRIFSLKLEDLVPAPPLIENMFRASLPF

>BmFTZ-F1—NP_001037528.2

MHEDAPKMSIAQSLAASTSQPKGDIVTEIPLEFAMSSMETKSIETTNVELKITYVDPTTGTGGEPGAYLPTAGTVCDQTDTKDVIEELCPVCGDKVSGYHYGLLTCESCKGFFKRTVQNKKVYTCVAERACHIDKTQRKRCPFCRFQKCLDVGMKLEAVRADRMRGGRNKFGPMYKRDRARKLQMMRQRQIAVQTLRGSLGDGGLVLGFGSPYTAVSVKQEIQIPQVSSLTSSPESSPGPALLGAQPQPPQPPPPPTHDKWEAHSPHSASPDAFTFDTQSNTAATPSSTAEATSTETLRVSPMIREFVQTVDDREWQNALFGLLQSQTYNQCEVDLFELMCKVLDQNLFSQVDWARNTVFFKYLKVDDQMKLLQDSWSVMLVLDHLHQRMHNGLPDETTLHNGQKFDLLCLGLLGVPSLADHFNELQNKLAELKFDVPDYICVKFMLLLNPEVRGIVNVKCVREGYQTVQAALLDYTLTCYPTIQDKFGKLVMVVPEIHALAARGEEHLYQRHCAGQAPTQTLLMEMLHAKRKS

>BmHR39—XP_012547940.1

APLQPHQRSLSMNRISVLTDNASLSLARRPSSPTNSAKSLALSDQGTMPVMAQTSIGEVQNLTIKKEILTDLKCEPSVGIDKLMGSLHGSPLLGRLPRVQSSASTDSAVHSMYTHSVYSSPSASPRASRHYTPSLSRNNSDASHSSCYSYSSEFSPTQSPVQGRHPHVMYREPAGVLSPAHDDDTEPTDDRLHHHQGISRQQLINSPCPICGDKISGFHYGIFSCESCKGFFKRTVQNRKNYMCLRGGNCPVTVATRKKCPACRFDKCLGCGMKLEAIREDRTRGGRSTYQCSYPLSGAASTGSLLSAHVSPTLRHATSLNCVNGPGSYSRGESSNSRVTPDIPPLLQEIMDVEHLWQYNESELNRLGSSPGSPSANPLLAASGITAQNSNPDFLADLCNIADHRLYKIVKWCKSLPLFKNISIDDQICLLINSWCELLVLSCCYRGVSTPGEVRVGGGRGITLHQSAKLGLTPCIERMLSFTDHLRRLRVDRYEYVALKVIVLLTSDAPELRESEKVRASQEKALAALQAYIATHSPGTPAKFGELLLRIPELQRTCQFFMQVGKEMLNPANKNKDGDGQSFNLLMELLRGDH
