# Supplementary material for: Molecular Characterization and Expression Profiling of Nuclear Receptor Gene Families in Oriental Fruit Fly, Bactrocera Dorsalis (Hendel)
Source: Insects. 2020 Feb 16;11(2):126. doi: 10.3390/insects11020126 (PMC7074177; doi:10.3390/insects11020126)
Supplement: Supplementary file 1 [file insects-11-00126-s001.zip › Table S3 List of specific primers for qRT-PCR NR.doc]

**Table S3 List of specific primers for qRT-PCR.**

| Gene | Primer | Primer sequence (5’-3’) | Nucleotide position ^a^ |
| --- | --- | --- | --- |
| *BdKNRL* | F | ATTCACATCATCAAGGGCTG | 874 - 1128 |
|  | R | ATGATTGTTGTTGAGCGCGT |  |
| *BdEGON* | F | CGGGCACATTACTCTCCACC | 1006 - 1192 |
|  | R | GCTCCTCTTGCGCATCATTG |  |
| *BdKNI* | F | AATCACTCTCACCCGTCTCC | 1153 - 1363 |
|  | R | GACGCGTATCCAACATTGCT |  |
| *BdE75* | F | GGTTCATTGATTCCTGGCAC | 4014 - 4225 |
|  | R | TTGGGCTAACAGAGTTTGGT |  |
| *BdE78* | F | GTACCGGGCTTTTGTGATTT | 795 - 997 |
|  | R | TGTTGGCGAAATTCACTAGC |  |
| *BdHR3* | F | CACGCGAACAATTTAGGGCA | 57 - 242 |
|  | R | CAGAACTTTGTGACCGGCG |  |
| *BdECR* | F | GCGAAGGTTGTAAGGGTTTC | 1471 - 1656 |
|  | R | CATAGCGCATTGATTCTCCG |  |
| *BdHR96* | F | CGAGAGTTGAAATTGGCCAG | 1809 - 2014 |
|  | R | CTTGACACATATCGCGGAAC |  |
| *BdHNF4* | F | GACAAAGACAAACGCAACCA | 441- 621 |
|  | R | CTCAATCTCGGCCTTAACCA |  |
| *BdUSP* | F | AAGCGGCTTAACATTGATCG | 1173 - 1358 |
|  | R | AAAAGTTGTGCAAATCGGCT |  |
| *BdHR78* | F | GAGTAACGAACGCAACAACA | 94 - 308 |
|  | R | CGGCAGAATTGACAACGATT |  |
| *BdTLL* | F | TTCGCGCAAAACTCATTCAT | 612 - 799 |
|  | R | ATTCCTTCCACGAATCCTCC |  |
| *BdHR51* | F | GCGAGTCTGTCCTTCCGAG | 667 - 867 |
|  | R | CACTGGCGGCTACTTCTTCT |  |
| *BdDSF* | F1 | ACCGAAACACCATCAGCAAC | 719 - 920 |
|  | R1 | ACCACTACCCGGCTTACAAT |  |
|  | F2 | GTCTGTTGTCGTGGGAAATG | 1203 - 1408 |
|  | R2 | GGTGATTCGAGTATGGGTGT |  |
|  | F3 | CTCTATGCAGCGCGTCAAAA | 1067 - 1281 |
|  | R3 | TCAAGCACTTAACCCAACGC |  |
| *BdHR83* | F1 | ATGTCGTGTGGAATGAGTGC | 588 - 738 |
|  | R1 | CTCAGGGCCAATTCTTTCCG |  |
|  | F2 | CGTCTATGATTGATGCCTGC | 625 - 844 |
|  | R2 | GCCAGTTCGTTTGTTGCATA |  |
|  | F3 | GGCTGCTCGTGTTTCTTCAA | 171 - 367 |
|  | R3 | GTGTTAAACTTTGGGTGCGC |  |
| *BdSVP* | F | AATACACACAACCAAAGCGG | 357 - 559 |
|  | R | TACTGCTCGCACCTAAACAA |  |
| *BdERR* | F | ACGTGCCCTGCTAATAATGA | 516 - 703 |
|  | R | GCAGTTGCATGGTTTGGTAT |  |
| *BdHR38* | F | GTTTGGGAGTAGGCATGGTA | 289 - 503 |
|  | R | GAGTTTACATGTGGCGGTTC |  |
| *BdFTZ-F1* | F | GCGAATACACAATGGCCTAC | 3026 - 3239 |
|  | R | TTGTTGACAATACCACGCAC |  |
| *BdHR39* | F | GCCCGACACATTCACCTATA | 1171 - 1387 |
|  | R | TATTCAGCTGCGAGACCTTT |  |
| *BdHR4* | F | GAGTCTGAATTTGCCACACC | 4436 - 4629 |
|  | R | CACCATATGACCGACATCCA |  |

F means forward, R means reverse.

a Nucleotide positions indicated for genes in Table1, where position #1 = the first nucleotide of the start codon.
